# Skeletal muscle reprogramming enhances reinnervation after peripheral nerve injury

**DOI:** 10.21203/rs.3.rs-3463557/v1

**Published:** 2024-01-05

**Authors:** Pihu Mehrotra, James Jablonski, John Toftegard, Yali Zhang, Shahryar Shahini, Jianmin Wang, Carey W Hung, Reilly Ellis, Gabriella Kayal, Nika Rajabian, Song Liu, Kelly Roballo, Susan B. Udin, Stelios T. Andreadis, Kirkwood E. Personius

**Affiliations:** 1 Department of Chemical and Biological Engineering, University at Buffalo, Buffalo, NY 14260, USA; 2 Department of Department of Rehabilitation Science, University at Buffalo, Buffalo, NY 14214, USA; 3 Department of Biomedical Engineering, University at Buffalo, NY, Buffalo, NY 14260, USA; 4 Department of Biostatistics and Bioinformatics, Roswell Park Comprehensive Cancer Center, Buffalo, NY 14203, USA; 5 Biomedical Affairs and Research, Edward Via College of Osteopathic Medicine, Blacksburg, VA 24060, USA; 6 Department of Biomedical Sciences and Pathobiology, Virginia Maryland College of Veterinary Medicine, Virginia Tech, Blacksburg, VA 24060, USA; 7 Department of Physiology and Biophysics, University at Buffalo, Amherst, NY 14203, USA; 8 Center of Excellence in Bioinformatics and Life Sciences, Buffalo, NY 14203, USA; 9 Center for Cell, Gene and Tissue Engineering (CGTE), University at Buffalo, Buffalo, NY 14260, USA

## Abstract

Peripheral Nerve Injuries (PNI) affect more than 20 million Americans and severely impact quality of life by causing long-term disability. The onset of PNI is characterized by nerve degeneration distal to the nerve injury resulting in long periods of skeletal muscle denervation. During this period, muscle fibers atrophy and frequently become incapable of “accepting” innervation because of the slow speed of axon regeneration post injury. We hypothesize that reprogramming the skeletal muscle to an embryonic-like state may preserve its reinnervation capability following PNI. To this end, we generated a mouse model in which NANOG, a pluripotency-associated transcription factor can be expressed locally upon delivery of doxycycline (Dox) in a polymeric vehicle. NANOG expression in the muscle upregulated the percentage of Pax7+ nuclei and expression of eMYHC along with other genes that are involved in muscle development. In a sciatic nerve transection model, NANOG expression led to upregulation of key genes associated with myogenesis, neurogenesis and neuromuscular junction (NMJ) formation, and downregulation of key muscle atrophy genes. Further, NANOG mice demonstrated extensive overlap between synaptic vesicles and NMJ acetylcholine receptors (AChRs) indicating restored innervation. Indeed, NANOG mice showed greater improvement in motor function as compared to wild-type (WT) animals, as evidenced by improved toe-spread reflex, EMG responses and isometric force production. In conclusion, we demonstrate that reprogramming the muscle can be an effective strategy to improve reinnervation and functional outcomes after PNI.

## Introduction

Peripheral Nerve Injuries (PNIs) refer to a range of sensorimotor impairments that occur when the peripheral nerves sustain damage or injury. These injuries are often the result of traumatic events like motor vehicle accidents, gunshot wounds, sports injuries, and combat wounds [1, 2]. PNIs predominantly affect young, healthy males with an average age of 40 years, leading to significant morbidity and disability [1, 3]. Surgical repair and reconstruction of damaged nerves pose significant clinical challenges, with less than half of the patients achieving normal sensory and motor function after nerve repair [4]. Patients with nerve injuries often experience chronic pain and persistent weakness, necessitating prolonged physical and occupational therapy for at least three months post-injury [5]. PNIs also impose a substantial economic burden on the US healthcare system, with an annual expenditure of approximately $150 billion, accounting for around 4.3% of total US health-related spending [1, 6].

Over the past decades, the use of nerve grafts to repair nerve injuries has been the gold standard in the field. Autologous sensory or motor nerves can be harvested from a patient and sutured at the site of injury to bridge the defect. However, the supply of the autologous nerve is limited, and most often requires a secondary surgery increasing chances of donor site morbidity. Additional complications of nerve grafts include formation of painful neuromas, infection, and sensory loss [7, 8]. Artificial nerve grafts or nerve conduits have been used as an alternative to autologous grafts and are commercially available and FDA approved [9]. The most commonly used materials include Type I Collagen [10], Chitosan [11, 12], Polyglycolic acid (PGA) [13], Poly(dl-lactide-ε-caprolactone; PCL) [14] and Polyvinyl Alcohol (PVA) [9]. While nerve conduits show satisfactory recovery for nerve injuries, most FDA-approved grafts are only suitable for defects shorter than 3.0 cm in length and are not effective for critical long-gap nerve defects [15, 16].

Nerve grafts can be decellularized to reduce immunogenicity while preserving the basement membrane and extracellular matrix (ECM) necessary for guiding axonal regeneration. However, acellular nerve grafts alone have not been as effective due to the absence of progenitor cells and Schwann cells that create a conducive microenvironment for regeneration [17]. Therefore, recent approaches focus on cellularizing grafts with specific cells that aid wound healing and nerve regeneration. Commonly used cell types for this purpose include Schwann cell presursors, Neural Stem cells, Bone Marrow-derived stem cells, Adipose-derived stem cells, and Skin-derived precursors. The use of these cells relies either on their ability to differentiate into functional Schwann cells at the injury site and/or secrete essential neurotrophic factors to facilitate axonal growth [18–20]. However, procuring these cells in a patient-specific manner to minimize immunogenic reactions has proven challenging [21].

As evident, most therapies to treat nerve injuries involve strategies to aid or accelerate nerve growth. The rate of human peripheral nerve regeneration is slow; approximately 1 inch per month [22]. As a result, skeletal muscle fibers remain denervated for long periods of time, leading to activation of atrophy related pathways, muscle wasting and ultimately loss of muscle function [23]. In fact, molecular changes in muscle function can be observed within a few hours of injury and denervation [24]. Consequently, by the time nerve endings reach the muscle for reinnervation, the muscle is severely atrophied and unable to ‘accept’ innervation [25]. Hence, finding ways to prevent muscle atrophy, or keeping the skeletal muscle in state that enables innervation might be crucial for better outcomes after PNIs.

Recent studies show that transient and partial reprogramming of cells or tissues using pluripotency factors can be an efficient way to enhance tissue regeneration while preventing teratomas [26]. In particular, Ocampo et al. showed that doxycycline (Dox)‐inducible transient expression of four pluripotency genes- Oct4, *Sox2, Klf4* and *c-Myc* (OSKM) erased several signs of aging, prolonged lifespan and enhanced the regenerative capacity of aged tissues. Notably, they observed an increase in Pax7+ muscle cells and enhanced skeletal muscle regeneration after cardiotoxin (CTX) injury [27]. Virus-free expression of pluripotency factors using intramuscular delivery of plasmid encoding for OSKM also improved skeletal muscle regeneration [28]. In our lab, we have extensively studied the effect of pluripotency-associated transcription factor NANOG in enhancing regenerative capacity of tissues. NANOG expression in bone marrow-derived Mesenchymal Stem Cells (BM-MSC) from adult human donors could restore their myogenic differentiation potential and contractile properties that are otherwise impaired by senescence [29]. NANOG expression also restored synthesis of ECM, especially Collagen type III (Col3) in senescent human MSCs and progeric fibroblasts via the TGFβ pathway [30]. Subsequently, we found that transient expression of NANOG using a Dox-inducible system could restore the myogenic differentiation potential of skeletal myoblasts [31]. NANOG expression reversed the aged phenotype of senescent human myoblasts, as seen by enhanced cellular proliferation, heterochromatin modifications and decrease in DNA damage [32]. Further, NANOG expression restored skeletal muscle regeneration in-vivo in progeric mice. We observed an increase in Pax7+ satellite cells and eMYHC+ myofibers after cardiotoxin injury in NANOG expressing progeric mice, along with an increase in skeletal muscle contraction force [33].

In all, we have established that NANOG expression enhanced skeletal muscle regeneration capacity and conferred a “young-like” state. Hence, we hypothesize that ectopic and transient NANOG expression may maintain the denervated skeletal muscle in a pro-regenerative state, preventing significant muscle atrophy and ultimately enabling better reinnervation and enhanced functional outcome post PNI.

## Results

### NANOG is expressed in skeletal muscle on Dox exposure through Elvax.

**Figure 1:**
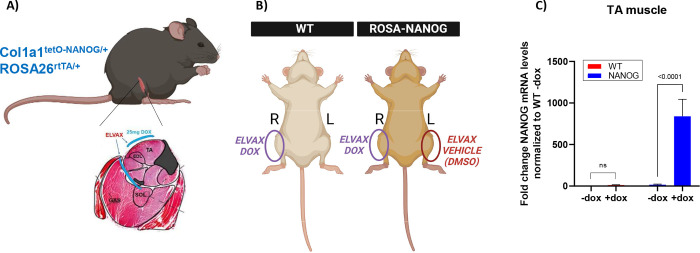
Mouse model for dox-inducible NANOG expression in skeletal muscle. **(A)** Localized NANOG expression in the muscle was achieved by subcutaneous implantation of the slow-release polymer Elvax (shown in blue) near the TA, EDL, Soleus and Gastrocnemius muscles. Schematic of muscle adapted from [107]. **(B)** Schematic of surgical implantation of Elvax in naïve uninjured WT and NANOG mice; the right leg of both received Elvax-DOX, while the left leg of NANOG mice received Elvax-DMSO (vehicle), and left leg of WT mice did not receive Elvax. **(C)** Fold change in relative mRNA levels of NANOG from isolated TA muscle 4 days after Elvax implantation in naïve uninjured WT and NANOG animals (normalized to WT; no Elvax).

To achieve skeletal muscle reprogramming using transient expression of NANOG, we used a transgenic mouse model, carrying the rtTA gene under the ROSA locus, and a cassette encoding for NANOG under the TetO promoter inserted in the Col1 locus. NANOG expression locally in the skeletal muscle was achieved using the slow-release polymer Elvax impregnated with 25mg Dox, that was implanted subcutaneously over the Tibialis Anterior (TA), Extensor Digitorum Longus (EDL), Soleus and Gastrocnemius muscles (Figure 1A). Given the ease of accessing the TA muscle for electrode placements in live mice for muscle function analysis, we limited our RNA and protein expression studies to the TA muscle.

To assess the extent of NANOG expression in skeletal muscle following Elvax placement, we first implanted Elvax impregnated with Dox (Elvax-Dox) on the right leg of naïve non-injured WT and NANOG animals. The left leg of naïve NANOG animals underwent vehicle implantation (Elvax-DMSO), while the left leg of naïve WT animals did not (Figure 1B). To assess NANOG expression upon dox administration, we performed RT-PCR with muscle tissues that were isolated after 4 days of Elvax placement. Indeed, the right TA muscle of NANOG mice showed ~800-fold increased mRNA levels of NANOG as compared to the left TA muscle, while NANOG expression remained unchanged in WT TA muscle on Dox administration (Figure 1C).

### NANOG expression reprograms skeletal muscle to a pro-regenerative state.

**Figure 2:**
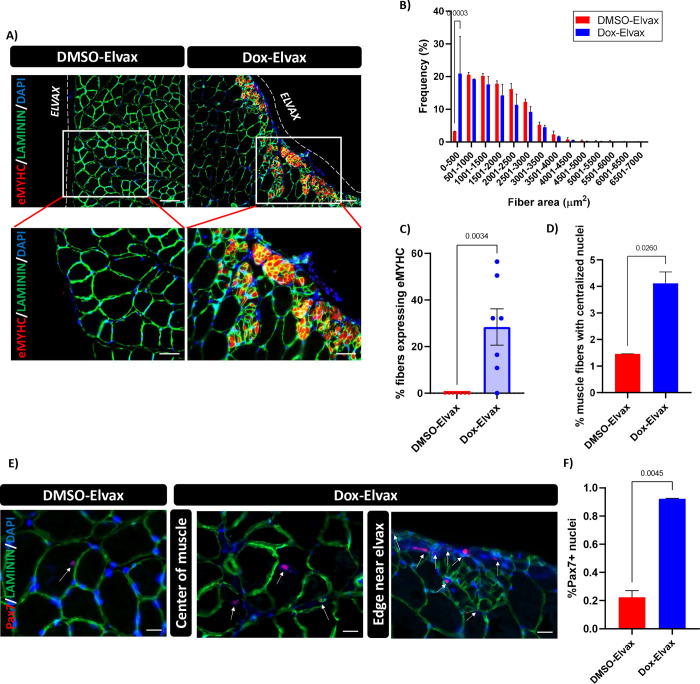
NANOG expression de-differentiates skeletal muscle to a pro-regenerative state. The left leg of naïve uninjured mice was implanted with Elvax-DOX for two weeks; the right leg was implanted with Elvax-DMSO and served as control for the Elvax placement. One week after removal of Elvax, the TA muscle was harvested for evaluation. **(A)** Immunostaining of TA muscle sections for eMYHC (red), Laminin (green) and DAPI (Blue). Scale bar- 100 μm, insets are higher magnification images with scale bar 50 μm. **(B)** Quantification of fiber area of TA muscle in mice with Elvax-DOX vs. Elvax-DMSO. **(C)** Quantification of small muscle fibers (less than 100 μm^2^) expressing eMYHC. **(D)** Percentage of centrally nucleated regenerative muscle fibers throughout the TA muscle. **(E)** Immunostaining for Pax7 (red), Laminin (green) and DAPI (Blue). Fields of view depicted representative images from the center of the muscle, and section adjacent to Elvax placement. Scale bar- 20μm. **(F)** Quantification for percentage of Pax7 positive nuclei throughout the TA muscle.

To assess the ability of transient NANOG expression to induce skeletal muscle rejuvenation, the right leg of naïve ROSA-NANOG animals was implanted with Elvax-Dox and the left leg with vehicle control Elvax-DMSO. After 2 weeks, the Elvax was removed and TA muscle was harvested 1 week later for evaluation of muscle structure, protein expression and markers of regeneration. We observed multiple small muscle fibers, especially close to the site of Elvax-Dox placement, suggesting skeletal muscle regeneration (Figure 2A). These fibers were smaller than 500 μm^2^ (Figure 2B) and expressed embryonic MYHC, an established marker of newly formed regenerative muscle fibers (Figure 2C). Further, NANOG expression resulted in ~3-fold increase in centrally nucleated fibers throughout the muscle tissue (Figure 2D). Immunostaining for the myogenic transcription factor Pax7 showed an increase in Pax7+ cells 1 week post Elvax removal. While the density of Pax7+ cells was higher at the edge of the muscle near the site of Elvax implantation, the percentage of Pax7+ cells was high throughout the NANOG expressing muscle (Figure 2E, F). Hence, transient NANOG expression induced fiber de-differentiation in an uninjured and non-denervated muscle, suggesting that it might reprogram the muscle to a state that enables re-innervation. Note, the placement of Elvax did not cause any injury to the muscle as evidenced by structurally intact muscle fibers (Figure 2A; DMSO-Elvax).

Previously, we have extensively studied the regenerative potential of the transcription factor NANOG in-vitro [32]. Human myoblasts were transduced with a lentiviral vector that encodes for NANOG under a tetracycline regulatable promoter (Supplementary Figure 1A). NANOG was expressed for 5 days by treating cells with Dox, and RNA was isolated thereafter to assess changes in transcription. Interestingly, we found that transient NANOG expression in human myoblasts enriched key signaling pathways crucial for nerve development, regeneration, and synapse formation, such as NGF signaling pathway, NMDA receptor activation, [34, 35], neurotrophin signaling and neurotransmitter receptor binding (Supplementary Figure 1B). Further, nerve associated genes such as NRG1, NRG2 and NGF were upregulated in human skeletal muscle cells that expressed NANOG. Additionally, NANOG significantly downregulated Atrogin-1 and Murf-1, key genes associated with skeletal muscle atrophy (Supplementary Figure 1C). These findings prompted us to examine whether NANOG expression in skeletal muscle promoted innervation, neuromuscular junction formation, and functional recovery after nerve injury.

### NANOG expression in denervated muscle upregulates key genes associated with skeletal muscle development and nerve growth.

**Figure 3:**
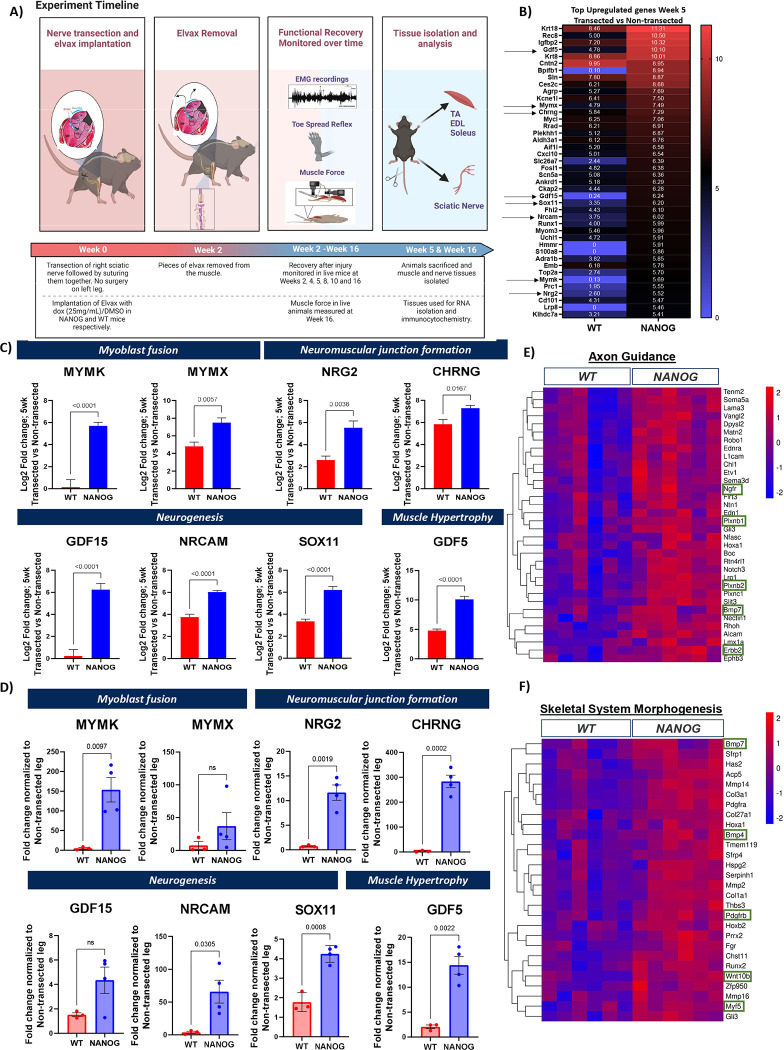
NANOG expression in skeletal muscle in a sciatic nerve injury model upregulates genes associated with skeletal muscle dedifferentiation, neurogenesis and nerve development. **(A)** Schematic depicting experimental procedure, timeline and experiments performed. **(B)** Top 42 upregulated genes in TA muscle on side of transection normalized to non-transected limb in WT and NANOG animals. Log2 fold change values are depicted within each cell. Genes of interest with respect to muscle or nerve development are highlighted by arrows. **(C)** Plot depicting log 2-fold change in selected genes from panel B associated with myoblast fusion (MYMK and MYMX), NMJ formation (NRG2 and CHRNG), neurogenesis (GDF15, NRCAM and Sox11) and muscle hypertrophy (GDF5) after transection, normalized to expression in non-transected limb of the same animal in WT and NANOG mice. **(D)** RT-PCR expression data from TA muscle for genes in Panel C on side of transection normalized to non-transected limb in WT and NANOG animals. **(E)** Heatmaps showing differentially expressed genes associated with gene ontology term Axon Guidance and **(F)** Skeletal System Morphogenesis. Only genes with statistically significant differences (padj<0.05) between WT and NANOG samples have been depicted. Genes of particular interest are highlighted within green boxes.

To understand the effect of skeletal muscle NANOG expression on peripheral nerve regeneration and synapse formation after nerve injury, we performed complete nerve transection of the right sciatic nerve followed by an end-to-end suture. At the same time, Elvax impregnated with Dox was implanted subcutaneously at the right skeletal muscle of NANOG and WT animals. The left sciatic nerve was left non-transected and served as an internal control for each animal. Elvax was removed after 2 weeks of nerve transection, and recovery was monitored over 16 weeks. Nerve and muscle tissues were isolated after 5 weeks and 16 weeks for RNA and protein isolation and immunocytochemistry analysis (Figure 3A).

Interestingly, RNA sequencing analysis on the TA muscle tissue 5 weeks post PNI revealed that the top 42 upregulated genes were common in both WT and NANOG animals. However, the extent of upregulation of these genes was higher in NANOG animals (Figure 3B). Specifically, we noticed that key genes related to embryonic muscle development and nerve regeneration were upregulated to a significantly greater extent upon NANOG expression. Myomarker (MYMK) and Myomixer (MYMX) govern muscle fusion and are essential genes involved in skeletal muscle regeneration during embryo development and in response to injury [36, 37]. Neureglin-2 (NRG2) is a nerve growth factor widely implicated in neuromuscular synapse formation [38, 39]. CHRNG encodes for an acetylcholine receptor isoform that is expressed in fetal and denervated but not in adult muscle and play a key role in formation of the motor endplate [40]. GDF15, NRCAM and SOX11 are involved in nerve development and subsequent synaptogenesis [41–44]. Lastly, GDF5 has been shown to suppress denervation related muscle atrophy [45]. While all these genes were upregulated after transection in both WT and NANOG mice, the extent of upregulation was significantly higher after NANOG expression (Figure 3C). We further confirmed this by RT-PCR analysis on TA muscle isolated from NANOG and WT animals at 5 weeks post transection. Indeed, expression of MYMK, NRG2, CHRNG, NRCAM, SOX11 and GDF5 was significantly upregulated in NANOG mice, while MYMX and GDF15 also increased although not significantly (Figure 3D).

Genes upregulated in the transected leg of NANOG animals were then annotated based on their function using Gene ontology (GO) enrichment analysis. Key genes associated with Axon guidance such as NGFR, Plxnb1, Plnxb2, Bmp7 and Erbb2 [46–49], as well as those implicated in skeletal system morphogenesis such as Bmp7, Bmp4, Pdgfrb, Wnt10b and Myf5 [50–53] were upregulated in NANOG compared to WT animals at 5 weeks after transection (Figure 3E, F).

In all, NANOG expression in the skeletal muscle upregulates genes that are implicated in three aspects of muscle function: (i) muscle development often during embryogenesis; (ii) nerve and synapse development; and (iii) skeletal muscle hypertrophy.

### NANOG expression enhanced gene pathways involved in ECM remodeling and nerve regeneration and downregulated pathways associated with muscle atrophy.

**Figure 4:**
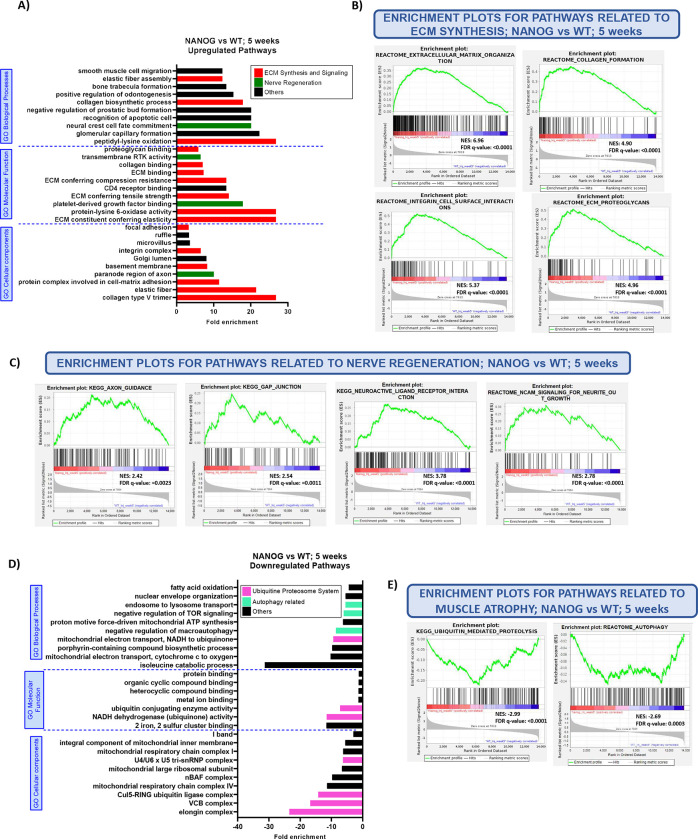
NANOG upregulates ECM organization and Nerve Regeneration pathways and downregulates atrophy-associated pathways 5 weeks after nerve transection. **(A)** Top 10 most highly enriched GO cellular components, molecular functions and biological processes. ECM: Extracellular Matrix, RTK: Receptor Tyrosine Kinase. **(B)** Enrichment plots from Reactome database depicting ECM synthesis and signaling associated pathways; ECM organization, Collagen formation, Integrin cell surface interactions and ECM proteoglycans. **(C)** Enrichment plots from KEGG and Reactome databases depicting nerve development pathways; Axon guidance, Gap junction, Neuroactive ligand receptor interaction and NCAM signaling for neurite outgrowth. **(D)** Top 10 most highly downregulated GO cellular components, molecular functions and biological processes. **(E)** Enrichment plots from KEGG and Reactome databases depicting Ubiquitin mediated proteolysis and Autophagy. NES: Normalized Enrichment Score.

To further evaluate the transcriptional effects of NANOG expression on the skeletal muscle, we performed Gene Set Enrichment Analysis (GSEA) and GO analysis using RNA isolated from the TA muscle at 5 weeks post transection. GO analysis revealed that pathways (cellular components, molecular functions, and biological processes) related to ECM synthesis and signaling, such as collagen formation and binding, and elastin assembly were among the top 10 most highly upregulated pathways in NANOG compared to WT mice at 5 weeks after nerve transection (Figure 4A; red bars). Further, multiple pathways related to nerve regeneration, such as Tyrosine Kinase Receptor activity, and platelet-derived growth factor (PDGF) signaling were also upregulated by NANOG (Figure 4A; green bars).

Interestingly the GO pathway “Synapse” was significantly enriched (Fold enrichment: 1.53, FDR: <0.0001) with NANOG (Supplemental Figure 2A). The key gene AGRN that has been extensively shown to be critical for formation of neuromuscular synapses [54–56] was upregulated by NANOG. Other genes critical for synapse formation and function such as Nlgn2 [57], Clcn5 [58], Sv2c [59], and Th [60] were also upregulated by NANOG (Supplemental Figure 2A; green arrows). Other significantly enriched GO terms included “Innervation”; (Fold enrichment: 5.19, FDR: 0.0047) (Supplemental Figure 2B), “Distal Axon” ; (Fold enrichment: 2.20, FDR: 0.0004) (Supplemental Figure 2C), “Cellular Response to PDGF Stimulus”; (Fold enrichment: 6.70, FDR: 0.0043) (Supplemental Figure 2D), “Semaphorin-Plexin signaling pathway involved in Axon Guidance”; (Fold enrichment: 8.37, FDR: 0.0024) (Supplemental Figure 2E), “Nerve Development”; (Fold enrichment: 3.65, FDR: 0.0009) (Supplemental Figure 2F), “Skeletal System Development”; (Fold enrichment: 2.84, FDR: <0.0001) (Supplemental Figure 2G) and “Collagen Fibril Organization”; (Fold enrichment: 11.63, FDR: <0.0001) (Supplemental Figure 2H). Most of these pathways have been previously implicated in aiding nerve regeneration and repair after PNI [61–63].

Gene Set Enrichment Analysis further confirmed this result; 80% of the top 20 most highly upregulated pathways by NANOG were associated with ECM organization and signaling upon nerve transection, while these pathways comprised only 10% of all the pathways upregulated in WT transected muscle (Supplementary Figure 3A–C). Specifically, Reactome database pathways correlated with ECM organization, collagen formation, integrin cell surface interactions, and ECM proteoglycans were significantly enriched by NANOG (Figure 4B). Additionally, KEGG database pathways pertaining to Axon Guidance, Gap junction and Neuroactive Ligand receptor Interaction, and the Reactome pathway NCAM signaling for Neurite Outgrowth was upregulated in the muscle of NANOG animals at 5 weeks after injury (Figure 4C). While we did observe upregulation of some ECM and nerve related pathways at 16 weeks after transection (Supplementary Figure 3D), the transcriptional changes were not as pronounced as on week 5. We did not observe any upregulation of embryonically relevant genes such as MYMK, MYMX and CHRNG at 16 weeks after transection, indicating that the muscle had approached its homeostatic state.

Further, we observed that NANOG expression downregulated key pathways and proteins associated with skeletal muscle atrophy. GO analysis revealed that the top 10 downregulated cellular components, molecular functions, and biological processes upon NANOG expression comprised of pathways associated with the Ubiquitin Proteosome System and Autophagy-related pathways (Figure 4D, Supplemental Figure 2I, J). GSEA enrichment analysis also revealed that the KEGG pathway correlated with Ubiquitin Mediated Proteolysis and Reactome database pathway correlated with Autophagy were significantly downregulated after 5 weeks of transection in NANOG mice (Figure 4E). Both pathways have been shown to play important roles in mediating muscle wasting [64–67].

These data suggest that NANOG expression alters the ECM landscape of the muscle, potentially aiding synaptogenesis and effective development and maturation of NMJs [68]. Further, essential neuronal signaling events that are important to establish synapses and aid nerve regeneration such as neuronal cell-adhesion molecules (NCAMs) [69] are also upregulated, while denervation associated muscle atrophy genes were downregulated by NANOG. Collectively, these transcriptomic changes suggest that NANOG expression might prime the skeletal muscle for enhanced re-innervation.

### NANOG expression enhanced neuromuscular junction formation.

**Figure 5:**
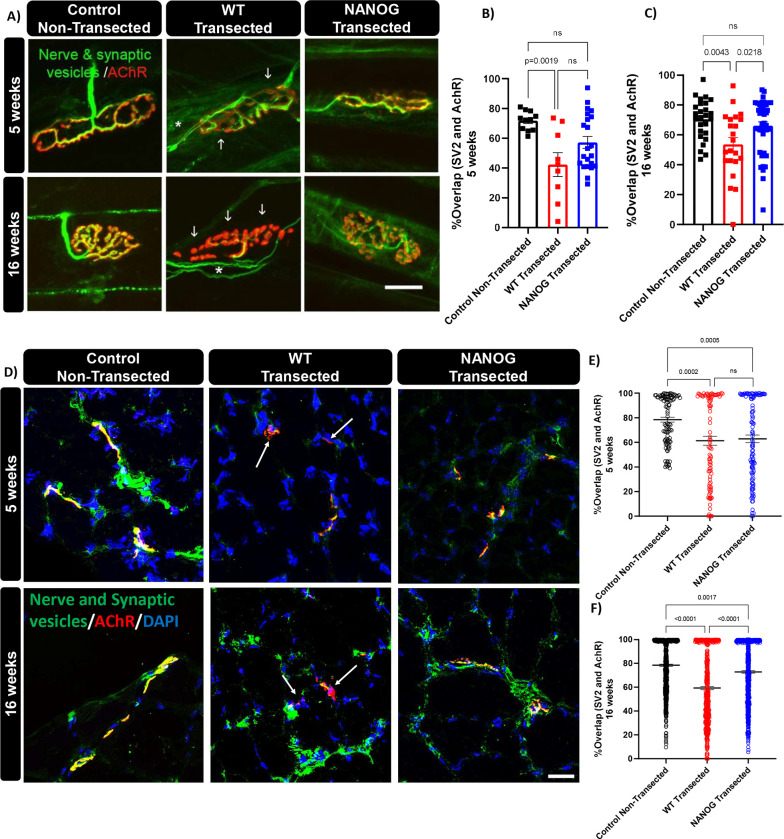
NANOG improves overlap between neuronal synaptic vesicles and muscle AChRs following transection. **(A)** Confocal images of NMJs 5 and 16 weeks after transection using whole mount immunocytochemistry. Presynaptic axons and synaptic vesicles (green) and postsynaptic AChRs (Red). Regions of overlap between pre- and post-synaptic regions are yellow. WT mice frequently demonstrate poor overlap between synaptic vesicles and AChRs (arrows). Multiple innervations were also seen in WT mice (asterisks). NANOG mice showed pre- vs. post-synaptic overlap similar to NMJs from control non-transected limbs. Scale bar = 20μm. Quantification for percentage of colocalization between synaptic vesicles and AChRs at **(B)** 5 weeks and **(C)** at 16 weeks. **(D)** Confocal images of NMJs 5 and 16 weeks after transection in muscle cross-sections. Presynaptic axons and synaptic vesicles (green) and postsynaptic AChRs (red). Regions of overlap between pre- and post-synaptic regions are yellow. WT mice frequently demonstrate poor overlap between synaptic vesicles and AChRs (arrows). NANOG mice showed overlap similar to NMJs from control non-transected limbs. Scale bar = 20μm. Quantification for percentage of colocalization between synaptic vesicles and AChRs at **(E)** 5 weeks and **(F)** 16 weeks after nerve injury.

To assess the effect of NANOG expression on structural changes in the reinnervated neuromuscular junction, we performed whole muscle immunohistochemistry in EDL and soleus muscles at 5 and 16 after sciatic nerve transection. The presynaptic axons (nerve filaments) and synaptic vesicles (SVs) were co-labelled, and their co-localization to postsynaptic AChRs was evaluated. In the muscles of control non-transected limb, the SVs were nearly completely colocalized with AChRs, and innervation of each NMJ was achieved by a single motor neuron (Figure 5A). Overlap between AChRs and SV2 was significantly decreased in WT animals as compared to non-transected controls at 5 weeks after nerve transection. Notably, we did not find any significant differences in overlap between non-transected controls and NANOG animals 5 weeks after transection (Control Non-transected: 71.8 ± 6.18%; NANOG: 57.2 ± 18.24%, WT: 42.28 ± 22.7 %, One-way ANOVA and Tukey’s post-hoc test; p=0.002; Figure 5A, B). This trend continued at 16 weeks post injury, with significantly decreased overlap in WT mice (53.6 ± 20.83 %) as compared to NANOG mice (66.12 ± 17.44 %, p=0.02) (Figure 5A, C). Interestingly, neuromuscular junctions of WT mice were frequently innervated by multiple axons at 5- and 16-weeks post injury (asterisk), indicating an immature state of innervation [70] (Figure 5A).

Whole mount immunohistochemistry only allows assessment of NMJs on the surface of the muscle fibers. To assess innervation throughout the entire muscle, we quantified colocalization between AChRs and SV2 in TA muscle cross-sections (Figure 5D). While there was no significant difference in overlap between WT and NANOG animals at 5 weeks post transection (Figure 5D, E), NANOG mice showed significantly improved overlap as compared to WT mice by 16 weeks (Control Non-transected: 78.47 ± 22.02 % NANOG: 72.91 ± 26.43 %; WT: 59.32 ± 30.81 %; One-way ANOVA and Tukey’s post-hoc test; p<0.0001; Figure 5D, F). Interestingly the extent of overlap was similar at 5 weeks and 16 weeks in WT mice, suggesting that structural reinnervation showed limited improvement. Taken together, these findings demonstrate that NANOG expression in the muscle enhanced NMJ formation and restored innervation, indicating efficient reinnervation after PNI.

### NANOG animals show trends of enhanced nerve myelination 5 weeks after injury.

Given the enhanced NMJ formation in NANOG animals post nerve transection, we were curious about the structural integrity and myelination levels of the sciatic nerve. GO pathway analysis revealed that key genes associated with gliogenesis (Fold enrichment: 2.68, FDR: <0.0001), glial cell migration (Fold enrichment: 4.66, FDR: 0.0020) and myelin assembly (Fold enrichment: 6.43, FDR: 0.0022) were upregulated by NANOG (Supplemental Figure 4A–C). Further, GSEA analysis revealed that the Reactome pathway “EGR2 and Sox10 mediated initiation of Schwann Cell Myelination” was also significantly enriched in NANOG animals 5 weeks post injury (Supplemental Figure 4D). To assess changes in myelination, we isolated the sciatic nerve from both non-transected and transected legs of WT and NANOG animals and stained for myelin using fluoromyelin red. We observed an increase (though not statistically significant) in fluoromyelin red stain intensity within the transected NANOG nerve (33.31 ± 6.28) compared to the transected WT nerve (24.02 ± 7.5). The area of increased myelination at the site of transection is indicated by the orange arrow within the encircled region of the transected NANOG nerve. The WT nerve showed decreased fluoromyelin at the transected site indicating impaired myelination. Furthermore, both WT and NANOG non-transected nerves showed similar levels of myelin, as evidenced by the comparable fluoromyelin red stain intensities (Supplemental Figure 4E–G). In all, this suggests that NANOG plays a role in remyelination of the transected nerve to some degree (Supplemental Figure 4G) and this may explain the enhanced recovery after PNI in NANOG animals.

### NANOG expression improved toe spread reflex, electromyographic (EMG) compound muscle action potential (CMAP) and muscle isometric force production.

**Figure 6:**
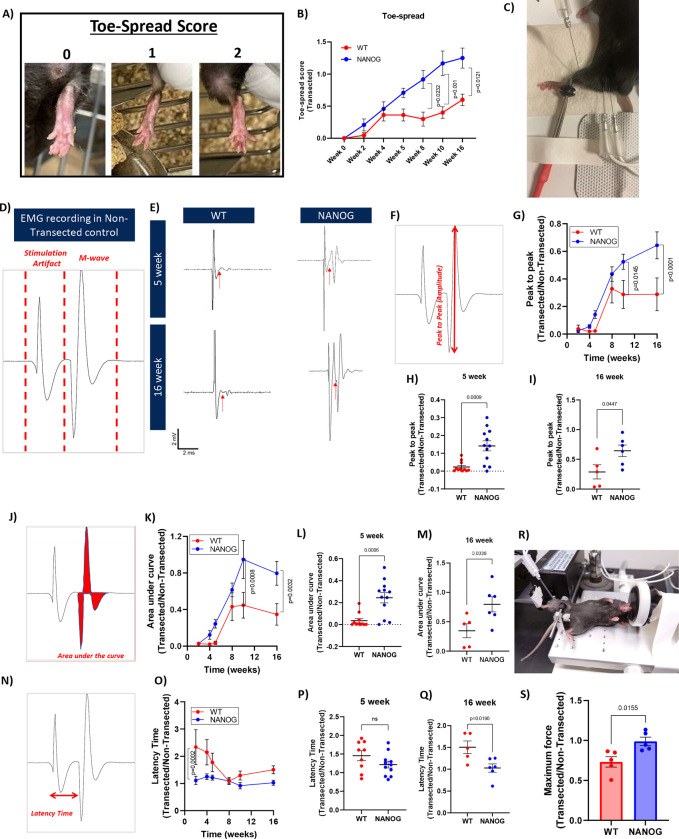
NANOG mice show enhanced functional recovery after nerve transection. **(A)** Depiction of mouse toe spread reflex and assigned scores. Scores range from 0 (no spread) to 2 (full spread of all toes). **(B)** Quantification of toe-spread reflex from Week 0 to Week 16 after nerve transection. **(C)** Setup for needle EMG recordings. The mouse was anesthetized and stimulating electrodes were placed on either side of the sciatic nerve above the site of transection. Recording electrodes were placed mid-belly in the TA muscle and around the ankle for differential recordings. **(D)** A typical EMG waveform recorded in non-transected control mice. The waveform includes a stimulation artifact followed by an CMAP excitation M-wave. **(E)** Representative waveforms from WT and NANOG transected limbs at 5 weeks and 16 weeks after injury. The start of M-wave is marked by red arrows. EMG recordings were completed in both the transected side and non-transected control limb. EMG for each animal was normalized to the control limb. **(F)** Schematic depicting the amplitude of the CMAP M-wave, calculated from the positive peak to the negative peak. **(G)** Quantification of EMG amplitude at 2, 4, 5, 8, 10, and 16 weeks after sciatic nerve transection in WT and NANOG mice. **(H)** EMG peak to peak at 5 weeks and **(I)** 16 weeks post nerve injury. **(J)** Schematic depicting the area of an M-wave, calculated as total area of the positive and negative CMAP M-wave curves. **(K)** Quantification of EMG area from the start of nerve transection at 2, 4, 5, 8, 10 and16 weeks after sciatic nerve transection in WT and NANOG mice. **(L)** CMAP M-wave area at 5 weeks and **(M)** 16 weeks post nerve injury. **(N)** Schematic of latency time, calculated as the time taken from the trigger of stimulus to the onset of M-wave depolarization response. **(O)** Quantification of EMG latency time at 2, 4, 5, 8, 10 and 16 weeks after sciatic nerve transection in WT and NANOG mice. **(P)** Latency time at 5 weeks and **(Q)** 16 weeks post nerve injury; n = 11–12 from 0–5 weeks and 5–6 from 8–16 weeks for control and NANOG, respectively. **(R)** Setup for muscle force measurement in live mice using the Aurora force transducer. The mouse was anesthetized, and its foot was secured to the footplate. Electrodes were placed subcutaneously over the TA muscle for stimulation, and the aggregate torque produced was recorded and quantified as force. **(S)** Maximum force recorded at stimulation frequency of 150 Hz from both transected side and non-transected control limb. Force in the transected side was normalized to the average force of the control non-transected limb for each condition; n = 5 for both WT and NANOG animals.

Functional recovery after nerve transection was investigated throughout 16 weeks of recovery. First, we ranked the toe-spread reflex in WT and NANOG animals after nerve transection. The return of toe spread reflex is a more sensitive measure of restored function than analysis of gait following sciatic nerve injury [71, 72]. The reflex was scored between 0–2; 0 indicating no spreading, 1 indicating intermediate spreading, and 2 indicating complete spreading of the toes (Figure 6A). The toe-spread score in WT mice improved up to Week 4 and was stalled thereafter. However, the toe-spread score in NANOG mice increased continually over 16 weeks and was significantly higher as compared to WT mice from 8 – 16 weeks (Two-way ANOVA and Tukey’s post-hoc test; p=0.0004; Figure 6B).

Next, we performed needle EMG recordings to measure the summated action potentials of all stimulated motor endplates within the TA muscle. Stimulating electrodes were placed on either side of the sciatic nerve proximal to the site of transection and the CMAP M-wave was recorded via an electrode placed at the mid-belly of the TA muscle (Figure 6C, D). As seen in the representative EMG CMAP, the transected leg of the NANOG mice produced a stronger and well-developed M-wave (arrows) resembling the recording in non-transected control. The EMG CMAP captured in WT mice showed weak muscle activation upon stimulation at both 5- and 16-weeks post nerve transection (Figure 6E). The CMAP waveforms were quantified based on amplitude (peak to peak) and area (under the waveform), both of which are indicators of synaptic strength [73] (Figure 6 F, J). EMG recordings were performed on transected and non-transected limbs for every animal at each time-point. Thus, all data was plotted as the ratio of transected to non-transected limb. By 5 weeks, WT mice showed limited reinnervation (2.3 ± 0.7% of non-transected control limb), while EMG CMAP amplitude significantly recovered to 14.2 ± 4.0% of the non-transected limb in ROSA-NANOG mice. EMG amplitude continued to improve to 64.3 ± 9.2% of the non-transected limb by 16 weeks in ROSA-NANOG mice, while recovery stalled at 21.7 ± 9.9% of the non-transected limb in WT mice (Two-way ANOVA and Tukey’s post-hoc test; p=0.0022; Figure 6G). In fact, the amplitude of M-wave in NANOG mice was improved by almost 7-fold as early as 5-weeks (Figure 6H), and the difference remained statistically significant at 16 weeks (Figure 6I). The CMAP area followed a similar trend (Figure 6J), reaching greater area in NANOG mice, but stagnating after 8 weeks in WT mice (Two-way ANOVA and Tukey’s post-hoc test; p=0.0105; Figure 6K). We found a 7-fold increase in area at 5 weeks (Figure 6L) and 2-fold increase at 16 weeks (Figure 6M) in NANOG animals.

We also quantified the onset latency time, which is determined by the time taken from the stimulus artifact to the onset of CMAP depolarization response [74] (Figure 6N). Increased latency time indicates a slower axon conduction speed of the fastest axons. Interestingly, NANOG mice had significantly lower latency time as early as 2 weeks after transection. The latency time in WT mice improved between 5 to 8 weeks post transection but did not improve further (Two-way ANOVA and Tukey’s post-hoc test; p>0.05; Figure 6O). While we did not find significant differences in latency time of WT and NANOG animals 5 weeks after transection (Figure 6P), the latency time of NANOG animals after 16 weeks was significantly lower (Figure 6Q). Taken together, these data suggest that NANOG animals exhibited improved reinnervation and formed functional synapses after sciatic nerve transection. While WT animals were capable of reinnervation, the recovery of EMG CMAP stagnated after 5–8 weeks.

Next, we measured isometric muscle force in transected and non-transected legs of live animals at 16 weeks after transection. Each animal was anesthetized while its foot was attached to the footplate fastened to a dual-mode lever. The TA muscle was stimulated by using subcutaneous EMG electrodes, and the aggregate torque produced was quantified as force generated by the muscle (Figure 6R). We found that the ratio of the force exerted by the transected over the non-transected limb was significantly higher in NANOG as compared to WT mice (NANOG: 98 ± 0.1 %; WT: 73 ± 0.13 %; Figure 6S). In all, we demonstrate that enhanced innervation is accompanied by enhanced muscle function, evidenced by complete recovery in isometric force production in NANOG mice.

## Discussion

Nerve injuries affect primarily young individuals and children, who potentially suffer for multiple decades after the injury [75]. Nerve injuries have a limited window of 18 months for sufficient reinnervation [76]. However, nerve lesions are often diagnosed after significant delay, leaving a short time for adequate treatment [77]. As a result, only partial recovery is achieved post PNI, and many patients experience chronic debilitating pain [78]. Most current approaches to treat nerve injuries rely on developing biomaterial scaffolds to bridge the nerve gap [79] or using Schwann cells to aid nerve regeneration [80] However, none of the current approaches address the challenges associated with denervation-associated atrophy of the muscle, often caused by slow rate of nerve growth and delayed medical intervention. Long-term muscle denervation has been shown to bring changes in the motor endplates making them non-permissive to axonal innervation and failing to establish a structurally sound NMJ [25].

In this study, we demonstrate a novel approach to improve functional outcomes after PNI. We use a transgenic mouse model to partially reprogram the skeletal muscle by expressing the embryonic transcription factor NANOG. Partial reprogramming has been used to reverse the hallmarks of aging while avoiding the risk of teratoma formation and preventing loss of tissue identity and function [81]. We have previously shown that transient NANOG expression could rejuvenate aged skeletal muscle and enhance muscle force [33]. In this study, we show that partial reprogramming of muscle by NANOG can de-differentiate it to an early-development like state, as seen by increased expression of eMYHC, a protein exclusively expressed during muscle development [82]. Transient NANOG expression also downregulated key genes governing muscle atrophy and increased transcription of genes associated with neurotropic factor signaling. Neurotropic factors such as BDNF have been shown to play a role in satellite cell proliferation and muscle regeneration [83], as well in maintaining mitochondrial function [84]. BDNF is secreted by both motor neurons and myofibers at the NMJ, and plays a role in postsynaptic maintenance, acetylcholine release, and motor neuron viability [85–87]. The neurotropic factor GDNF is expressed in Soleus and EDL muscles during early myogenesis [88]. Further, the neurotropin receptors p75, TrkA, TrkB, TrkC and Ret are expressed abundantly in the skeletal muscle [89], and cluster around the pre- and post-synaptic sides of the NMJ [90, 91]. Hence, we hypothesized that NANOG expression might be priming the muscle to a state that is conducive to innervation.

Indeed, we found that NANOG induced ECM remodeling of the muscle might play a role in improving innervation. Previously, we have shown that NANOG expression restores ECM synthesis in senescent MSCs [92]. Studies on human fetal sclera fibroblasts confirm the role of NANOG in improving Col1 expression [93]. ECM proteins play a crucial role in NMJ formation and synaptogenesis during development. The ECM proteins secreted by each individual myofiber for the synaptic basal lamina mark the location of NMJs and induce synaptic localization [94]. Key ECM proteins that comprise the synaptic cleft include laminin and collagen [95], both of which are upregulated by NANOG. Hence, this ECM remodeling might play a role in creating a permissive environment for axons to grow and form synapses. Enhanced synaptogenesis is confirmed by enhanced colocalization of presynaptic neuronal vesicles, and postsynaptic acetylcholine receptors in NANOG animals. In WT animals, the lack of essential ECM proteins could lead to immature synapses, as evidenced by innervation by multiple axons 5 weeks after injury, many of which might fail to mature over time.

Most importantly, we show that NANOG expression in the muscle enhances motor function, as evidenced by improved toe-spread reflex and EMG CMAP. WT mice show maximum recovery between 5 to 8 weeks after nerve transection but don’t improve thereafter. Interestingly, NANOG expression initiates early recovery after nerve transection. Improvements in toe-spread reflex and EMG CMAP are observed as early as 5 weeks, and recovery keeps improving throughout 16 weeks post nerve transection. It is interesting to note that recovery evaluated based on all three parameters- toe-spread, EMG CAMP and synapse formation evidenced by overlap between pre- and post-synaptic regions convey a similar trend in both WT and NANOG mice. While WT mice showed limited improvement in all these parameters after 5 weeks, NANOG mice showed continued improvements throughout 16 weeks post transection. This early recovery and continued improvement in muscle function may be attributed to enhanced transcription of key genes necessary for synaptogenesis. While both WT and NANOG animals upregulated genes associated with NMJ formation and neurogenesis, and downregulated atrophy-related genes, the changes were significantly enhanced by partial reprogramming with NANOG. Though it is difficult to comment on why this might be so, it is very well established that partial reprogramming initiated by embryonic factors (a.k.a. Yamanaka factors) leads to genome-wide epigenetic modifications influencing gene transcription [81, 96].

In summary, we clearly demonstrate that NANOG-induced muscle reprogramming can prime adult muscle to accept new synapses and enhance recovery after PNI, without loss of cellular identity. Further studies are needed to decode the exact mechanisms that might be aiding enhanced synaptogenesis. Knowledge of these mechanisms and signaling events that mediate this process is crucial to identify druggable targets and design small molecules that mimic NANOG and can be pharmacologically administered to patients following injury. In this study, NANOG was expressed in the periphery at the time of injury. However, clinical interventions can often only be provided after the injury has taken place. Hence, further work is needed to determine the time window after injury wherein NANOG expression leads functional improvements. Moreover, techniques of reprogramming the muscle can be used in conjunction with nerve conduits, autologous grafts, and stem cells to enhance recovery after PNI. In conclusion, we show for the first time that it is possible to reprogram muscle into an innervation permissive state, leading to enhanced outcomes after nerve injury.

## Materials and Methods

### 1. Generation of ROSA-NANOG Transgenic mice

Transgenic mice that could express NANOG on exposure to doxycycline (C57BL/6; Col1a1 tetO-Nanog/+; ROSA26rtTA/rtTA) were provided by the laboratory of Dr. Manuel Serrano at The Barcelona Institute of Science and Technology, Spain [97]. The mice carry the M2-rtTA gene inserted within the Rosa26 locus, and a cassette containing NANOG cDNA under the Dox-responsive promoter (tetO) is inserted downstream of the Col1a1 locus. Mice were bred and genotyped using polymerase chain reaction (PCR) of tail DNA using the primers in Table 1 for DNA amplification. Wild-type (WT) mice that did not express NANOG served as controls. Experiments were performed with equal number of male and female mice (WT: 5 females and 6 males; NANOG: 5 females and 6 males) that were 9–10 months old at the time of transection. The mice were kept in a controlled environment where they experienced a 12-hour cycle of light and darkness from 6:00 to 18:00. The room temperature was maintained at 22°C, and they had unrestricted access to food and water. The humidity levels were maintained between 30% and 70%. All research involving animals followed approved protocols from the Institutional Animal Care and Use Committee (IACUC) of the University at Buffalo. These protocols adhered to the Animal Welfare Act, Public Health Service Policy on humane care and use of laboratory animals, and other relevant federal statutes and regulations governing animal experimentation.

### 2. Elvax preparation and implantation

For slow release of doxycycline (Dox), Elvax sheets were prepared as previously described [35]. Briefly, Elvax 40W beads (obtained as a gift from DuPont) were subjected to 3–4 rounds of washing in 95% ethanol with continuous stirring over the course of one week. Subsequently, they were dried using filter paper. In a glass culture tube, 100 mg of these beads were dissolved in 900 μl of methylene chloride. For drug incorporation, Dox (25mg/100μl; Catalog # D9891, Sigma-Aldrich, St. Louis, MO), or saline was dissolved in a solution of 1% fast green in DMSO. The Elvax mixture was then vortexed at a medium speed for 3 minutes and poured onto a glass slide with a cut piece of Parafilm serving as a spacer. The slide, along with a second slide clamped on top, was placed on powdered dry ice. The Elvax was then exposed to a temperature of 70° for 2–5 days before being shifted to 20°C. Before use, the Elvax was briefly rehydrated using saline.

To implant the Elvax, 9–10 months old mice were anesthetized using isoflurane. An incision was made in the lateral skin of the distal hindlimb, and the fascial plane between the anterior and posterior compartments was cut. Two rectangular pieces of Elvax measuring 3×4 mm were placed in the hindlimb. One was place subcutaneously over the (TA) muscle. The second was placed between the anterior and posterior compartments near the gastrocnemius and soleus muscles (Figure 1A). After suturing the incision, the mice were returned to their cages following a warming period. The right leg received Elvax impregnated with dox, while the left leg did not receive any Elvax. The Elvax was left in place for 2 weeks and removed thereafter in a second surgery. In some control experiments the right leg was implanted with Dox-Elvax and the left leg with Dox-DMSO to verify that Elvax placement did not damage the muscle directly.

### 3. Nerve transection

Mice were anesthetized using isoflurane. The right sciatic nerve for each animal was completely transected 3mm proximal to the trifurcation and immediately repaired with 10–0 micro suture (Polypropylene-Sharpoint, eSutures.com). Elvax impregnated with dox was implanted on the right hindlimb to both ROSA-NANOG and WT mice in the same surgery. Surgery was conducted in 6 male and 5 female WT mice, and 6 male and 5 female N+ mice. Recovery was monitored over several weeks. After 5 weeks of nerve injury, 5 mice from each condition were euthanized, and muscle and nerve samples were collected for analysis. After 16 weeks, the remaining mice were euthanized, and tissues were collected for analysis.

### 4. Toe-spread test

The toe-spread reflex test was conducted as described previously [34]. The mice were carefully covered with a cloth and lifted by their tail, allowing their hind leg digits to fully extend and spread. The reflex response was evaluated and scored as follows: 0 indicated no spreading of the toes, 1 represented intermediate spreading, and 2 indicated complete spreading (refer to Figure 6A). The test was conducted at Weeks 0, 2,4,5,8,10, and 16 weeks.

### 5. EMG recordings

Mice were anesthetized by isoflurane. EMG recordings were made using procedures modified from Arnold et al, 2015 [98]. The sciatic nerve was stimulated at the proximal hind limb above the site of transection using two 28 G monopolar electrodes (DTM Series, Electrode Store). Stimulation was provided by a Grass S88 (Quincy, MA) using square-wave pulses of 0.1 ms duration and intensity ranging from 1–10 mA. Recordings were made using one fine ring electrode (reference) placed around the ankle and a second 28 G monopolar electrode (active) placed mid-belly in the TA. A ground electrode was placed around the tail to allow for differential amplification of the CMAP waveform (BDA-H-4, WPI, Inc). Signals were acquired digitally and analyzed using Spike2 software (CED, Inc, Cambridge, UK). Stimulation intensity was increased until there was no more increase in CMAP amplitude. To ensure supramaximal stimulation, stimulation intensity was increase to ~120% of maximal response. EMG recording were made from both the left control non-denervated TA and on the side of transection (Right). EMG CMAP results are presented as ratio of the recordings from left and right sides.

### 6. Protein isolation and Western blot

TA muscles were weighed, and 9–10 mg of the muscle was cut for protein isolation. Muscles lysed in RIPA buffer (Catalog # 89900, Thermo Fisher Scientific, Waltham, MA). containing 1X Halt protease and phosphatase inhibitor (Catalog # 78442, Thermo Fisher Scientific). TA muscle tissues were homogenized by bead disruption using Bead Lysis tubes (Catalog # GREENR5-RNA, Next Advance, Troy, NY) using the Bullet Blender (Stellar Scientific, Baltimore, MD), which was chilled using dry-ice. Lysates were centrifuged and 1X Blue loading dye (Cell Signaling, Danvers, MA) and 1X DTT reducing agent (Cell Signaling) were added. Protein was denatured by incubation at 95 °C for 5 min and proteins were loaded at 45 μg per lane and were separated in 10% acrylamide gels (Thermo Fisher Scientific) by SDS-polyacrylamide gel electrophoresis based on their molecular weight. After transferring proteins to nitrocellulose membranes (Bio-Rad, Hercules, California) using the Trans-Blot Turbo Transfer System (Bio-Rad), the membranes were blocked in 5% (w/v) non-fat dry milk in blocking buffer for 1 h at room temperature. Subsequently, membranes were incubated overnight at 4 °C with antibodies listed in Table 2. Membranes were incubated for 1 h at room temperature with Anti-rabbit IgG HRP linked (Catalog # 7074, Cell Signaling). Finally, the protein bands were visualized using horseradish peroxidase-conjugated secondary antibodies and a chemiluminescence kit (Cell Signalling) according to the manufacturer’s instructions. Luminescent blots were imaged using ChemiDoc^™^ Touch Imaging System (Bio-Rad).

### 7. RNA isolation from tissues and qPCR

TA muscles were weighed, and 9–10 mg of the muscle was cut for RNA isolation. RNA was isolated using RNeasy Fibrous Tissue Mini Kit (Catalog # 74704, Qiagen, Valencia, CA) as per manufacturer’s instructions. cDNA was synthesized using the Superscript III cDNA Synthesis Kit (Thermo Fisher Scientific). For real-time PCR analysis, 1 μg of cDNA was used per reaction, and the SYBR Green Kit (Bio-Rad) was employed. The specific primers for the real-time PCR were listed in Table 3. The qPCR cycle determination was performed using Bio-Rad Software CFX Manager Ver 3.1.

### 8. Whole mount immunocytochemistry

The soleus and EDL muscles were placed in a dish coated with Sylgard and soaked in saline solution. Connective tissue was removed from the muscles, and they were fixed by immersing them in 4% paraformaldehyde solution at pH 7.4 for 15 minutes. Subsequently, the muscles were transferred to microcentrifuge tubes and washed three times with PBS. They were then exposed to rhodamine-conjugated-α-bungarotoxin (α-BTX; Catalog # T1175, Life Technologies, Grand Island, NY) at a concentration of 10% for 20 minutes, followed by three additional rinses with PBS. To permeabilize the tissue, the muscles were immersed in −20°C methanol for 5 minutes and then rinsed three times with PBS.

Next, the muscles were blocked in a solution containing 2% bovine serum albumin, 0.1% sodium azide, and 0.2% Triton-X100 in saline for 60 minutes. They were then incubated overnight at room temperature with primary antibodies against SV2 (synaptic vesicles) and 2H3 (neurofilament) (Table 2). The staining was visualized using a secondary antibody, Alexa Fluor 488 donkey anti-mouse (RRID: AB_2556542, Catalog # R37114; Life Technologies). Finally, the muscles were filleted into two halves and pressed flat on the slide via coverslip and Vectashield mounting medium. Staining was observed using conventional epifluorescence microscopy (63× oil objective, BX51; Olympus) and confocal microscopy (63× oil objective, LSM 510 Meta NLO; Zeiss). The extent of colocalization between synaptic vesicles and acetylcholine receptors was analyzed using Fiji (ImageJ).

### 9. Tissue embedding and immunostaining.

TA, EDL and Soleus muscles were isolated, and connective tissue, if any, was carefully removed. The muscle was gently dried and then immersed in OCT embedding medium (Sakura Finetek, Torrance, CA). Next the tissues were transferred to a container containing dry ice and 2-Methylbutane (Sigma-Aldrich) to freeze the tissues. For staining, tissue sections were cut to 10μm thickness using a cryostat (Leica CM1950, Buffalo Grove, IL) at −20°C, and placed on positively charged glass slides (Stellar Scientific). Sections were stored at −80 °C.

For immunostaining for eMYHC, Pax7 and Laminin, tissues were prepared by first washing them three times in PBS to remove the OCT embedding medium. Subsequently, they were fixed at room temperature, using 4% paraformaldehyde for 10 minutes. The slides were washed thrice. Next, they were immersed in R-Buffer A (Electron Microscopy Sciences, Hatfield, PA) for antigen retrieval, with the temperature raised to 95°C for 20 minutes, followed by gradual cooling. To quench endogenous peroxidase activity, the slides were treated with Tyramide H2O2 solution (Alexa Fluor^™^ 555 Tyramide SuperBoost^™^ Kit, Thermo Fisher Scientific) for 30 minutes. This was followed by blocking with a mixture of 5% (w/v) goat serum and 5% (w/v) BSA in PBS for 1 hour, followed by Tyramide Blocking Buffer for another hour, and mouse IgG blocking reagent (MOM, Vectors Lab, Burlingame, CA) for an additional hour, following the manufacturer’s protocol. The tissue sections were then incubated overnight at 4°C with primary antibodies (Table 2) diluted in MOM diluent. The next day, the samples were washed thrice with PBS and stained using the Tyramide kit goat anti-mouse secondary antibody, according to the Tyramide kit protocol. Finally, the samples were stained with Alexa Fluor 568, 488, or 647 conjugated goat anti-rabbit or goat anti-mouse secondary antibodies for 1 hour at room temperature. Finally, the cells were stained with Hoechst 33342 nuclear dye (1:1000 dilution in PBS, Thermo Fisher Scientific) for 5 min at RT, followed by three washes in PBS. The slides were coverslipped using ProLong^™^ Diamond Antifade Mountant (Thermo Fisher Scientific).

For immunostaining for SV2, 2H3 and ACHRs, slides were washed and fixed as described previously [34]. They were then blocked in a solution containing 2% bovine serum albumin, 0.1% sodium azide, and 0.2% Triton-X100 in saline for 60 minutes at room temperature. This was followed by incubation with primary antibodies for SV2 and 2H3 for 60 minutes at room temperature. The slides were then washed thrice and incubated with α-BTX at a concentration of 10% for 20 minutes, followed by three additional rinses with PBS. Finally, the slides were stained with Hoechst 33342, washed with PBS thrice, and mounted for imaging.

### 10. RNA sequencing and pathway analysis

The global gene expression profiles were characterized by next generation RNA sequencing using Illumina platform. To this end, total RNA was isolated for all conditions using RNeasy Mini Kit and quality control analysis was performed by RNA gel and Agilent Fragment Analyzer. Sequencing libraries were prepared as per standard Illumina protocols (Illumina Stranded Total RNA Prep with Ribo-Zero Plus), quality checked, and quantified by Kapa Biosystems qPCR. The multiplexed libraries were sequenced in pair-end (2 X 50 bp) on the NovaSeq 6000 at 300 pM with 1% loading control.

Sequencing reads passed quality filter from Illumina RTA were first processed using FASTQC (v0.10.1) for sequencing base quality control. Then sample reads were aligned to the human reference genome (GRCm38) and GENCODE (version 22) annotation database using STAR2 [99]. Second round of QC using RSeQC [100] was applied to mapped bam files to identify potential RNASeq library preparation problem. Gene level raw counts were obtained using Subread [101] package. Differential gene expression analysis was performed using DESeq2 [102] and pathway analysis was performed with the Gene Set Enrichment Analysis (GSEA) method (4.2) [103], and the Gene ontology Resource [104, 105]. The GSEA tool was chosen to run the analysis using the normalized gene count data that pre-filtered the low count genes. Pathway analysis was run against MSigDB, a collection of annotated and curated gene set repositories offered by the developer of GSEA (Broad Institute MIT and Harvard). This particular run used C2 of version 7.5 collection, containing 2318 gene sets from various well-known and up-to-date pathway databases such as BioCarta, KEGG and Reactome among others.

### 11. In-vivo muscle isometric force measurement

Muscle isometric force was measured in live animals as described previously [32]. In short, mice were anesthetized using isoflurane. Next, the skin above the TA was carefully shaved, and the mice were placed on a heated stage maintained at 37 °C by circulating warm water. The knee of the mice was clamped using a knee clamp attached to the stage, and the leg was secured onto a footplate connected to a servomotor (1300 A: 3-in-1 Whole Animal System—Mouse; Aurora Scientific, CA). Two 28 G monopolar needle electrodes were inserted subcutaneously medial and lateral to the TA muscle. The optimal position for muscle contraction was determined by adjusting the distance between the footplate and knee and stimulating the muscle with a single electrical pulse (25 mA, 0.2 ms pulse width; previously optimized conditions). Once the muscle force ceased to increase, the position was considered the best for muscle contraction. Subsequently, Tetanic force was measured by stimulating the muscle with a 500 ms duration and 0.2 ms pulse width at frequencies ranging from 10 to 200 Hz (10, 20, 35, 50, 65, 80, 100, 150, 200 Hz), with a 2-minute interval between each stimulation. At the end of the experiments, the mice were returned to their cages with a warming pad and monitored until they regained consciousness and exhibited normal behavior. The data obtained were analyzed using 611 A Dynamic Muscle Analysis (DMA) software. Maximum Force was recorded at the stimulation frequency of 150 Hz and plotted as such.

### 12. Nerve labeling

The methodology was based on previously published protocol [106]. Briefly, nerves were isolated at 5 weeks and 16 weeks after PNI. The nerve was placed into Trump’s fixative for 24 hr, followed by 24 hr in 4% glutaraldehyde. Samples were embedded in Tissue-Tek^®^ O.C.T. Compound and frozen at −20°C and long-term storage at −80 °C. Tissue was cut at 10 μm thickness using a cryostat and stained with FluoroMyelin^™^ Red (Catalog # F34652; Thermo Fisher Scientific), and sections were placed on treated glass slides (Catalog # 9951LPLUS; Thermo Fisher Scientific). Nerves were imaged over ECLIPSE Ti2 Series NIKON Inverted Microscope. Main grey intensity, which reflected the quantity of myelination in the nerve, was quantified by individuals blinded to experimental conditions.

### 13. Imaging and Image analysis

The Zeiss Axio Observer Z1 inverted microscope (LSM 510; Zeiss, Oberkochen, Germany) equipped with an ORCA-ER CCD camera (Hamamatsu, Japan) was used to acquire immunocytochemistry and immunohistochemistry images. Confocal images were acquired using the Stellaris 5 confocal microscope (Leica).

Image analysis was performed using Fiji (ImageJ). For analyzing colocalization between synaptic vesicles and acetylcholine receptors, using the colocalization threshold tool. Area of interest was selected over the staining for α-BTX, after normalizing by subtracting fluorescence intensity in the region of interest over the AChRs from fluorescence intensity in adjacent control areas of the same size. Percentage of overlap of selected region of interest (ROI) in α-BTX channel with SV2 channel was plotted. To analyze fiber size, each muscle fiber was manually selected, and area was measured. To analyze number of fibers positive for eMYHC, positively stained fibers were manually counted. To quantify the number of Pax7+ nuclei, all nuclei that co-stained for Pax7 and Hoechst 33342 (DAPI) were manually counted.

### 14. Statistical analysis

All data was organized and collected as Microsoft Excel spreadsheet and statistical analysis was performed using GraphPad Prism^®^8 Software. Comparisons among multiple groups and time-points were performed using two-way ANOVA followed by Tukey’s post hoc test or Sidak’s multiple comparisons test. Comparisons among more than two samples were performed using one-way ANOVA followed by Tukey’s post hoc test. We used paired Student’s t-test to compare data depicting only two conditions. Comparisons having p-value<0.05 was considered statistically significant. All data was plotted as Mean ± SEM.

## Supplementary Material

Supplement 1

## Figures and Tables

**Table 1:** Primer sequences for genotyping

Target gene	Sequence 5’ ➝ 3’
NANOG primers	CCCTCCATGTGTGACCAAGG-common
	GCACAGCATTGCGGACATGC-wt
	GCAGAAGCGCGGCCGTCTGG-tg
rtTA primers	AAAGTCGCTCTGAGTTGTTAT-common
	GCGAAGAGTTTGTCCTCAACC-mut F
	GGAGCGGGAGAAATGGATATG-mut R

**Table 2:** List of antibodies

Antibody (Clone#)	Catalog #	# Dilution	Company
Pax7	AB-528428	ICC: 1:10	Developmental Studies Hybridoma Bank
eMyHC	Sc-53091	ICC: 1:100	Santa Cruz Biotechnology
Laminin	L9393	ICC: 1:200	Sigma-Aldrich
SV2	AB_2315387	WHI: 1:20 ICC: 1:100	Developmental Studies Hybridoma Bank
2H3	AB_531793	WHI: 1:10 ICC: 1:200	Developmental Studies Hybridoma Bank
NANOG	Ab80892	WB: 1:1000	Abcam

WHI: Whole mount Immunocytochemistry; ICC: Immunocytochemistry; WB: Western Blot

**Table 3:** List of primers (mouse)

Target Gene	Forward 5’ → 3’	Reverse 5’ → 3’
*NANOG*	TCTTCCTGGTCCCCACAGTTT	GCAAGAATAGTTCTCGGGATGAA
*MYMK*	TTCCTCCCGACAGTGAGCAT	GCACAGCACAGACAAACCAG
*MYMX*	CTGAGCTGTCTGCTCTTTGT	TCTCCTTCCTCTGGGAGTG
*NRG2*	GGATGGCAAGGAACTCAACC	TCGGCCTCACAGACGTACT
*CHRNG*	CTGATGCGAAACTACGACCCC	CCTCTCGTTCATTCAGGGAGATG
*GDF15*	CTGGCAATGCCTGAACAACG	GGTCGGGACTTGGTTCTGAG
*NRCAM*	GAGTGCCCCTGATTCTCTTCC	GATGGTTGGAGGTTGTACCAG
*SOX11*	CGACGACCTCATGTTCGACC	GACAGGGATAGGTTCCCCG
*GDF5*	CCATCACACCCCACGAATACA	GCTGCTGTTACCTCCCTTTCT

## References

[1] Taylor C.A., , The incidence of peripheral nerve injury in extremity trauma. American journal of physical medicine & rehabilitation, 2008. 87(5): p. 381–385. 18334923 10.1097/PHM.0b013e31815e6370

[2] Rivera J.C., Glebus G., and Cho M., Disability following combat-sustained nerve injury of the upper limb. The bone & joint journal, 2014. 96(2): p. 254–258. 24493193 10.1302/0301-620X.96B2.31798

[3] Bergmeister K.D., , Acute and long-term costs of 268 peripheral nerve injuries in the upper extremity. PloS one, 2020. 15(4): p. e0229530. 32251479 10.1371/journal.pone.0229530 PMC7135060

[4] Grinsell D. and Keating C., Peripheral nerve reconstruction after injury: a review of clinical and experimental therapies. BioMed research international, 2014. 2014. 10.1155/2014/698256 PMC4167952 25276813

[5] Padovano W.M., , Incidence of nerve injury after extremity trauma in the United States. Hand, 2022. 17(4): p. 615–623. 33084377 10.1177/1558944720963895 PMC9274890

[6] Conway P.H., Factors associated with increased US health care spending: implications for controlling health care costs. Jama, 2017. 318(17): p. 1657–1658. 29114814 10.1001/jama.2017.16802

[7] Rbia N. and Shin A.Y., The role of nerve graft substitutes in motor and mixed motor/sensory peripheral nerve injuries. The Journal of hand surgery, 2017. 42(5): p. 367–377. 28473159 10.1016/j.jhsa.2017.02.017

[8] Liu W., , Allotransplanted neurons used to repair peripheral nerve injury do not elicit overt immunogenicity. PLoS One, 2012. 7(2): p. e31675. 22347502 10.1371/journal.pone.0031675 PMC3276507

[9] Kehoe S., Zhang X., and Boyd D., FDA approved guidance conduits and wraps for peripheral nerve injury: a review of materials and efficacy. Injury, 2012. 43(5): p. 553–572. 21269624 10.1016/j.injury.2010.12.030

[10] Wangensteen K.J. and Kalliainen L.K., Collagen tube conduits in peripheral nerve repair: a retrospective analysis. Hand, 2010. 5(3): p. 273–277. 19937145 10.1007/s11552-009-9245-0 PMC2920394

[11] Haastert-Talini K., , Chitosan tubes of varying degrees of acetylation for bridging peripheral nerve defects. Biomaterials, 2013. 34(38): p. 9886–9904. 24050875 10.1016/j.biomaterials.2013.08.074

[12] Marcol W., , Reduction of post-traumatic neuroma and epineural scar formation in rat sciatic nerve by application of microcrystallic chitosan. Microsurgery, 2011. 31(8): p. 642–649. 22009638 10.1002/micr.20945

[13] Navissano M., , Neurotube^®^ for facial nerve repair. Microsurgery, 2005. 25(4): p. 268–271. 15937888 10.1002/micr.20128

[14] Den Dunnen W., , Poly (DL-lactide-ϵ-caprolactone) nerve guides perform better than autologous nerve grafts. Microsurgery: Official Journal of the International Microsurgical Society and the European Federation of Societies for Microsurgery, 1996. 17(7): p. 348–357. 10.1002/(SICI)1098-2752(1996)17:7<348::AID-MICR2>3.0.CO;2-C 9379881

[15] Kaplan H.M., Mishra P., and Kohn J., The overwhelming use of rat models in nerve regeneration research may compromise designs of nerve guidance conduits for humans. Journal of Materials Science: Materials in Medicine, 2015. 26: p. 1–5. 10.1007/s10856-015-5558-4 PMC4545171 26296419

[16] Kornfeld T., Vogt P.M., and Radtke C., Nerve grafting for peripheral nerve injuries with extended defect sizes. Wiener Medizinische Wochenschrift (1946), 2019. 169(9): p. 240. 30547373 10.1007/s10354-018-0675-6 PMC6538587

[17] Saheb-Al-Zamani M., , Limited regeneration in long acellular nerve allografts is associated with increased Schwann cell senescence. Experimental neurology, 2013. 247: p. 165–177. 23644284 10.1016/j.expneurol.2013.04.011 PMC3863361

[18] Patel N.P., Lyon K.A., and Huang J.H., An update–tissue engineered nerve grafts for the repair of peripheral nerve injuries. Neural regeneration research, 2018. 13(5): p. 764. 29862995 10.4103/1673-5374.232458 PMC5998615

[19] Fathi S.S. and Zaminy A., Stem cell therapy for nerve injury. World Journal of Stem Cells, 2017. 9(9): p. 144. 29026460 10.4252/wjsc.v9.i9.144 PMC5620423

[20] Yi S., , Application of stem cells in peripheral nerve regeneration. Burns & trauma, 2020. 8: p. tkaa002. 32346538 10.1093/burnst/tkaa002 PMC7175760

[21] Liu M., , Stem cells in the treatment of neuropathic pain: research progress of mechanism. Stem Cells International, 2020. 2020. 10.1155/2020/8861251 PMC7785341 33456473

[22] Höke A., A (heat) shock to the system promotes peripheral nerve regeneration. The Journal of clinical investigation, 2011. 121(11). 10.1172/JCI59320 PMC3204849 21965324

[23] Kostrominova T.Y., Skeletal Muscle Denervation: Past, Present and Future. 2022, MDPI. p. 7489. 10.3390/ijms23147489 PMC9316613 35886838

[24] Tang H., , A histone deacetylase 4/myogenin positive feedback loop coordinates denervation-dependent gene induction and suppression. Molecular biology of the cell, 2009. 20(4): p. 1120–1131. 19109424 10.1091/mbc.E08-07-0759 PMC2642751

[25] Sakuma M., , Lack of motor recovery after prolonged denervation of the neuromuscular junction is not due to regenerative failure. European Journal of Neuroscience, 2016. 43(3): p. 451–462. 26332731 10.1111/ejn.13059 PMC4738060

[26] de Lázaro I., Cossu G., and Kostarelos K., Transient transcription factor (OSKM) expression is key towards clinical translation of in vivo cell reprogramming. EMBO molecular medicine, 2017. 9(6): p. 733–736. 28455313 10.15252/emmm.201707650 PMC5452046

[27] Ocampo A., , In vivo amelioration of age-associated hallmarks by partial reprogramming. Cell, 2016. 167(7): p. 1719–1733. e12. 27984723 10.1016/j.cell.2016.11.052 PMC5679279

[28] de Lázaro I., , Non-viral, tumor-free induction of transient cell reprogramming in mouse skeletal muscle to enhance tissue regeneration. Molecular Therapy, 2019. 27(1): p. 59–75. 30470628 10.1016/j.ymthe.2018.10.014 PMC6318817

[29] Han J., , Nanog reverses the effects of organismal aging on mesenchymal stem cell proliferation and myogenic differentiation potential. Stem Cells, 2012. 30(12): p. 2746–2759. 22949105 10.1002/stem.1223 PMC3508087

[30] Rong N., , Restoring extracellular matrix synthesis in senescent stem cells. The FASEB Journal, 2019. 33(10): p. 10954. 31287964 10.1096/fj.201900377R PMC6766659

[31] Shahini A., , NANOG restores the impaired myogenic differentiation potential of skeletal myoblasts after multiple population doublings. Stem Cell Research, 2018. 26: p. 55–66. 29245050 10.1016/j.scr.2017.11.018

[32] Shahini A., , Ameliorating the hallmarks of cellular senescence in skeletal muscle myogenic progenitors in vitro and in vivo. Science Advances, 2021. 7(36): p. eabe5671. 34516892 10.1126/sciadv.abe5671 PMC8442867

[33] Rajabian N., , Methionine adenosyltransferase2A inhibition restores metabolism to improve regenerative capacity and strength of aged skeletal muscle. Nature Communications, 2023. 14(1): p. 886. 10.1038/s41467-023-36483-3 PMC9935517 36797255

[34] Personius K.E., , Blockage of neuromuscular glutamate receptors impairs reinnervation following nerve crush in adult mice. Frontiers in Cellular Neuroscience, 2022. 16: p. 1000218. 36212695 10.3389/fncel.2022.1000218 PMC9535682

[35] Personius K.E., Slusher B.S., and Udin S.B., Neuromuscular NMDA receptors modulate developmental synapse elimination. Journal of Neuroscience, 2016. 36(34): p. 8783–8789. 27559162 10.1523/JNEUROSCI.1181-16.2016 PMC6601906

[36] Chen B., , The regulatory role of Myomaker and Myomixer–Myomerger–Minion in muscle development and regeneration. Cellular and Molecular Life Sciences, 2020. 77: p. 1551–1569. 31642939 10.1007/s00018-019-03341-9 PMC11105057

[37] Bi P., , Fusogenic micropeptide Myomixer is essential for satellite cell fusion and muscle regeneration. Proceedings of the National Academy of Sciences, 2018. 115(15): p. 3864–3869. 10.1073/pnas.1800052115 PMC5899482 29581287

[38] Lee K.-H., , Bidirectional signaling of neuregulin-2 mediates formation of GABAergic synapses and maturation of glutamatergic synapses in newborn granule cells of postnatal hippocampus. Journal of Neuroscience, 2015. 35(50): p. 16479–16493. 26674872 10.1523/JNEUROSCI.1585-15.2015 PMC6605516

[39] Ponomareva O., , Stimulation of acetylcholine receptor transcription by neuregulin-2 requires an N-box response element and is regulated by alternative splicing. Neuroscience, 2005. 134(2): p. 495–503. 15961242 10.1016/j.neuroscience.2005.04.028

[40] Robinson K.G., , Neuromotor synapses in Escobar syndrome. American Journal of Medical Genetics Part A, 2013. 161(12): p. 3042–3048. 10.1002/ajmg.a.36154 PMC5600816 24038971

[41] Strelau J., , Progressive postnatal motoneuron loss in mice lacking GDF-15. Journal of Neuroscience, 2009. 29(43): p. 13640–13648. 19864576 10.1523/JNEUROSCI.1133-09.2009 PMC3320210

[42] Jiang W.-W., , Emerging roles of growth differentiation factor‐15 in brain disorders. Experimental and Therapeutic Medicine, 2021. 22(5): p. 1–11. 10.3892/etm.2021.10705 PMC8456456 34594407

[43] Demyanenko G.P., , Neural cell adhesion molecule NrCAM regulates Semaphorin 3F-induced dendritic spine remodeling. Journal of Neuroscience, 2014. 34(34): p. 11274–11287. 25143608 10.1523/JNEUROSCI.1774-14.2014 PMC4138338

[44] Wang Y., , Transcription factor Sox11 is essential for both embryonic and adult neurogenesis. Developmental Dynamics, 2013. 242(6): p. 638–653. 23483698 10.1002/dvdy.23962

[45] Hitachi K., Nakatani M., and Tsuchida K., Long non-coding RNA myoparr regulates GDF5 expression in denervated mouse skeletal muscle. Non-coding RNA, 2019. 5(2): p. 33. 30965639 10.3390/ncrna5020033 PMC6631233

[46] Wood S., Pritchard J., and Sofroniew M., Re-expression of nerve growth factor receptor after axonal injury recapitulates a developmental event in motor neurons: differential regulation when regeneration is allowed or prevented. European Journal of Neuroscience, 1990. 2(7): p. 650–657. 12106299 10.1111/j.1460-9568.1990.tb00454.x

[47] Deng S., , Plexin-B2, but not Plexin-B1, critically modulates neuronal migration and patterning of the developing nervous system in vivo. Journal of Neuroscience, 2007. 27(23): p. 6333–6347. 17554007 10.1523/JNEUROSCI.5381-06.2007 PMC6672150

[48] Liu S., , Overexpression of bone morphogenetic protein 7 reduces oligodendrocytes loss and promotes functional recovery after spinal cord injury. Journal of Cellular and Molecular Medicine, 2021. 25(18): p. 8764–8774. 34390115 10.1111/jcmm.16832 PMC8435414

[49] Kwon Y.K., , Activation of ErbB2 during wallerian degeneration of sciatic nerve. Journal of Neuroscience, 1997. 17(21): p. 8293–8299. 9334404 10.1523/JNEUROSCI.17-21-08293.1997 PMC6573732

[50] Winbanks C.E., , The bone morphogenetic protein axis is a positive regulator of skeletal muscle mass. Journal of Cell Biology, 2013. 203(2): p. 345–357. 24145169 10.1083/jcb.201211134 PMC3812980

[51] Sugg K.B., , Inhibition of platelet-derived growth factor signaling prevents muscle fiber growth during skeletal muscle hypertrophy. FEBS letters, 2017. 591(5): p. 801–809. 28129672 10.1002/1873-3468.12571 PMC5352504

[52] von Maltzahn J., , Wnt signaling in myogenesis. Trends in cell biology, 2012. 22(11): p. 602–609. 22944199 10.1016/j.tcb.2012.07.008 PMC3479319

[53] Yamamoto M., , Loss of MyoD and Myf5 in skeletal muscle stem cells results in altered myogenic programming and failed regeneration. Stem cell reports, 2018. 10(3): p. 956–969. 29478898 10.1016/j.stemcr.2018.01.027 PMC5918368

[54] Misgeld T., , Agrin promotes synaptic differentiation by counteracting an inhibitory effect of neurotransmitter. Proceedings of the National Academy of Sciences, 2005. 102(31): p. 11088–11093. 10.1073/pnas.0504806102 PMC1182450 16043708

[55] Samuel M.A., , Agrin and synaptic laminin are required to maintain adult neuromuscular junctions. 2012. 10.1371/journal.pone.0046663 PMC3463559 23056392

[56] Ksiazek I., , Synapse loss in cortex of agrin-deficient mice after genetic rescue of perinatal death. Journal of Neuroscience, 2007. 27(27): p. 7183–7195. 17611272 10.1523/JNEUROSCI.1609-07.2007 PMC6794585

[57] Wu M., , Impairment of inhibitory synapse formation and motor behavior in mice lacking the NL2 binding partner LHFPL4/GARLH4. Cell reports, 2018. 23(6): p. 1691–1705. 29742426 10.1016/j.celrep.2018.04.015

[58] Qi Y., , Identification of chloride channels CLCN3 and CLCN5 mediating the excitatory Cl− currents activated by sphingosine-1-phosphate in sensory neurons. Frontiers in molecular neuroscience, 2018. 11: p. 33. 29479306 10.3389/fnmol.2018.00033 PMC5811518

[59] Dunn A.R., , Synaptic vesicle glycoprotein 2C (SV2C) modulates dopamine release and is disrupted in Parkinson disease. Proceedings of the National Academy of Sciences, 2017. 114(11): p. E2253–E2262. 10.1073/pnas.1616892114 PMC5358362 28246328

[60] Nagatsu T., , The role of tyrosine hydroxylase as a key player in neuromelanin synthesis and the association of neuromelanin with Parkinson’s disease. Journal of Neural Transmission, 2023. 130(5): p. 611–625. 36939908 10.1007/s00702-023-02617-6 PMC10121510

[61] Yamazaki T., , Activation of MAP kinases, Akt and PDGF receptors in injured peripheral nerves. Journal of the Peripheral Nervous System, 2009. 14(3): p. 165–176. 19909480 10.1111/j.1529-8027.2009.00228.x

[62] Funa K. and Sasahara M., The roles of PDGF in development and during neurogenesis in the normal and diseased nervous system. Journal of Neuroimmune Pharmacology, 2014. 9: p. 168–181. 23771592 10.1007/s11481-013-9479-z PMC3955130

[63] Koropouli E. and Kolodkin A.L., Semaphorins and the dynamic regulation of synapse assembly, refinement, and function. Current opinion in neurobiology, 2014. 27: p. 1–7. 24598309 10.1016/j.conb.2014.02.005 PMC4122587

[64] Attaix D., , The ubiquitin–proteasome system and skeletal muscle wasting. Essays in biochemistry, 2005. 41: p. 173–186. 16250905 10.1042/EB0410173

[65] Mitch W.E. and Goldberg A.L., Mechanisms of muscle wasting—the role of the ubiquitin–proteasome pathway. New England journal of medicine, 1996. 335(25): p. 1897–1905. 8948566 10.1056/NEJM199612193352507

[66] Sandri M., Autophagy in health and disease. 3. Involvement of autophagy in muscle atrophy. American Journal of Physiology-Cell Physiology, 2010. 298(6): p. C1291–C1297. 20089936 10.1152/ajpcell.00531.2009

[67] Bodine S.C., , Akt/mTOR pathway is a crucial regulator of skeletal muscle hypertrophy and can prevent muscle atrophy in vivo. Nature cell biology, 2001. 3(11): p. 1014–1019. 11715023 10.1038/ncb1101-1014

[68] Zhang W., Liu Y., and Zhang H., Extracellular matrix: An important regulator of cell functions and skeletal muscle development. Cell & bioscience, 2021. 11: p. 1–13. 33789727 10.1186/s13578-021-00579-4 PMC8011170

[69] Washbourne P., , Cell adhesion molecules in synapse formation. Journal of Neuroscience, 2004. 24(42): p. 9244–9249. 15496659 10.1523/JNEUROSCI.3339-04.2004 PMC6730099

[70] Turney S.G. and Lichtman J.W., Reversing the outcome of synapse elimination at developing neuromuscular junctions in vivo: evidence for synaptic competition and its mechanism. PLoS biology, 2012. 10(6): p. e1001352. 22745601 10.1371/journal.pbio.1001352 PMC3383738

[71] Bozkurt A., , Aspects of static and dynamic motor function in peripheral nerve regeneration: SSI and CatWalk gait analysis. Behavioural brain research, 2011. 219(1): p. 55–62. 21168447 10.1016/j.bbr.2010.12.018

[72] Ma C.H.E., , Accelerating axonal growth promotes motor recovery after peripheral nerve injury in mice. The Journal of clinical investigation, 2011. 121(11). 10.1172/JCI58675 PMC3223863 21965333

[73] Mills K.R., The basics of electromyography. Journal of Neurology, Neurosurgery & Psychiatry, 2005. 76(suppl 2): p. ii32–ii35. 15961866 10.1136/jnnp.2005.069211 PMC1765694

[74] Kouzaki K., , Increases in M-wave latency of biceps brachii after elbow flexor eccentric contractions in women. European journal of applied physiology, 2016. 116: p. 939–946. 26994769 10.1007/s00421-016-3358-2

[75] Aman M., , Peripheral nerve injuries in children—prevalence, mechanisms and concomitant injuries: a major trauma center’s experience. European journal of medical research, 2023. 28(1): p. 1–7. 36907874 10.1186/s40001-023-01082-x PMC10008601

[76] Birch R. and Achan P., Peripheral nerve repairs and their results in children. Hand clinics, 2000. 16(4): p. 579–595. 11117049

[77] Boecker A.H., , Evaluation of MR-neurography in diagnosis and treatment in peripheral nerve surgery of the upper extremity: A matched cohort study. Microsurgery, 2022. 42(2): p. 160–169. 34931723 10.1002/micr.30846

[78] Eser F., , Etiological factors of traumatic peripheral nerve injuries. Neurology India, 2009. 57(4): p. 434. 19770544 10.4103/0028-3886.55614

[79] Bellamkonda R.V., Peripheral nerve regeneration: an opinion on channels, scaffolds and anisotropy. Biomaterials, 2006. 27(19): p. 3515–3518. 16533522 10.1016/j.biomaterials.2006.02.030

[80] Nemati Mahand S., , Application of stem cells, growth factors, small molecules, and biological macromolecules on nerve regeneration: a review and future direction. International Journal of Polymeric Materials and Polymeric Biomaterials, 2023: p. 1–33.

[81] Puri D. and Wagner W., Epigenetic rejuvenation by partial reprogramming. BioEssays, 2023. 45(4): p. 2200208. 10.1002/bies.202200208 36871150

[82] Agarwal M., , Myosin heavy chain-embryonic regulates skeletal muscle differentiation during mammalian development. Development, 2020. 147(7): p. dev184507. 32094117 10.1242/dev.184507 PMC7157585

[83] Clow C. and Jasmin B.J., Brain-derived neurotrophic factor regulates satellite cell differentiation and skeltal muscle regeneration. Molecular biology of the cell, 2010. 21(13): p. 2182–2190. 20427568 10.1091/mbc.E10-02-0154 PMC2893983

[84] Ahuja P., , Muscle-generated BDNF (brain derived neurotrophic factor) maintains mitochondrial quality control in female mice. Autophagy, 2022. 18(6): p. 1367–1384. 34689722 10.1080/15548627.2021.1985257 PMC9225428

[85] Arosio B., , Sarcopenia and Cognitive Decline in Older Adults: Targeting the Muscle–Brain Axis. Nutrients, 2023. 15(8): p. 1853. 37111070 10.3390/nu15081853 PMC10142447

[86] Personius K.E. and Parker S.D., TrkB expression at the neuromuscular junction is reduced during aging. Muscle & Nerve, 2013. 47(4): p. 532–538. 23180620 10.1002/mus.23616

[87] Kulakowski S.A., Parker S.D., and Personius K.E., Reduced TrkB expression results in precocious age-like changes in neuromuscular structure, neurotransmission, and muscle function. Journal of applied physiology, 2011. 111(3): p. 844–852. 21737823 10.1152/japplphysiol.00070.2011

[88] Wehrwein E.A., Roskelley E.M., and Spitsbergen J.M., GDNF is regulated in an activity-dependent manner in rat skeletal muscle. Muscle & Nerve: Official Journal of the American Association of Electrodiagnostic Medicine, 2002. 26(2): p. 206–211. 10.1002/mus.10179 12210384

[89] Sakuma K. and Yamaguchi A., The recent understanding of the neurotrophin’s role in skeletal muscle adaptation. Journal of Biomedicine and Biotechnology, 2011. 2011. 10.1155/2011/201696 PMC3179880 21960735

[90] Fu A.K., , Muscle-derived neurotrophin-3 increases the aggregation of acetylcholine receptors in neuron–muscle co-cultures. Neuroreport, 1997. 8(18): p. 3895–3900. 9462462 10.1097/00001756-199712220-00011

[91] Wells D.G., , Neurotrophins regulate agrin-induced postsynaptic differentiation. Proceedings of the National Academy of Sciences, 1999. 96(3): p. 1112–1117. 10.1073/pnas.96.3.1112 PMC15359 9927702

[92] Shahini A., , NANOG restores contractility of mesenchymal stem cell-based senescent microtissues. Tissue Engineering Part A, 2017. 23(11–12): p. 535–545. 28125933 10.1089/ten.tea.2016.0494 PMC5467120

[93] Li X., , NANOG improves type I collagen expression in human fetal scleral fibroblasts. Archives of Biological Sciences, 2019. 71(1): p. 63–70.

[94] Singhal N. and Martin P.T., Role of extracellular matrix proteins and their receptors in the development of the vertebrate neuromuscular junction. Developmental neurobiology, 2011. 71(11): p. 982–1005. 21766463 10.1002/dneu.20953 PMC3472639

[95] Yurchenco P.D., Amenta P.S., and Patton B.L., Basement membrane assembly, stability and activities observed through a developmental lens. Matrix Biology, 2004. 22(7): p. 521–538. 14996432 10.1016/j.matbio.2003.10.006

[96] Simpson D.J., Olova N.N., and Chandra T., Cellular reprogramming and epigenetic rejuvenation. Clinical Epigenetics, 2021. 13(1): p. 1–10. 34488874 10.1186/s13148-021-01158-7 PMC8419998

[97] Piazzolla D., , Lineage-restricted function of the pluripotency factor NANOG in stratified epithelia. Nature communications, 2014. 5(1): p. 4226. 10.1038/ncomms5226 24979572

[98] Arnold W.D., , Electrophysiological motor unit number estimation (MUNE) measuring compound muscle action potential (CMAP) in mouse hindlimb muscles. JoVE (Journal of Visualized Experiments), 2015(103): p. e52899. 10.3791/52899 PMC4676269 26436455

[99] Dobin A., , STAR: ultrafast universal RNA-seq aligner. Bioinformatics, 2013. 29(1): p. 15–21. 23104886 10.1093/bioinformatics/bts635 PMC3530905

[100] Rosenthal R., , DeconstructSigs: delineating mutational processes in single tumors distinguishes DNA repair deficiencies and patterns of carcinoma evolution. Genome biology, 2016. 17(1): p. 1–11. 26899170 10.1186/s13059-016-0893-4 PMC4762164

[101] Liao Y., Smyth G.K., and Shi W., The Subread aligner: fast, accurate and scalable read mapping by seed-and-vote. Nucleic acids research, 2013. 41(10): p. e108–e108. 23558742 10.1093/nar/gkt214 PMC3664803

[102] Love M.I., Huber W., and Anders S., Moderated estimation of fold change and dispersion for RNA-seq data with DESeq2. Genome biology, 2014. 15(12): p. 1–21. 10.1186/s13059-014-0550-8 PMC4302049 25516281

[103] Subramanian A., , Gene set enrichment analysis: a knowledge-based approach for interpreting genome-wide expression profiles. Proceedings of the National Academy of Sciences, 2005. 102(43): p. 15545–15550. 10.1073/pnas.0506580102 PMC1239896 16199517

[104] Ashburner M., , Gene ontology: tool for the unification of biology. Nature genetics, 2000. 25(1): p. 25–29. 10802651 10.1038/75556 PMC3037419

[105] Thomas P.D., , PANTHER: Making genome-scale phylogenetics accessible to all. Protein Science, 2022. 31(1): p. 8–22. 34717010 10.1002/pro.4218 PMC8740835

[106] Roballo K.C., , Long-term neural regeneration following injury to the peroneal branch of the sciatic nerve in sheep. European Journal of Neuroscience, 2020. 52(10): p. 4385–4394. 32449561 10.1111/ejn.14835

[107] Lee J.J., , Systematic interrogation of angiogenesis in the ischemic mouse hind limb: vulnerabilities and quality assurance. Arteriosclerosis, Thrombosis, and Vascular Biology, 2020. 40(10): p. 2454–2467. 32787524 10.1161/ATVBAHA.120.315028 PMC7505144

