# Supplementary material for: Skeletal muscle reprogramming enhances reinnervation after peripheral nerve injury
Source: Res Sq. 2024 Jan 5:rs.3.rs-3463557. Preprint. [Version 1] doi: 10.21203/rs.3.rs-3463557/v1 (PMC10802751; doi:10.21203/rs.3.rs-3463557/v1)

Supplemental Figure 1: NANOG expression upregulates genes and pathways related to neurogenesis in human myoblasts and downregulates key atrophy genes. (A) Schematic depicting the lentiviral vector encoding for NANOG in a Tet-on construct. LTR: Long Terminal Repeat. **(B)** Enrichment plots from Reactome and KEGG databases depicting nerve development associated pathways; NGF signaling via TrkA from the plasma membrane, Post NMDA receptor activation events, Neurotrophin signaling pathway and Neurotransmitter receptor binding and

downstream transmission in the post-synaptic cell. NES: Normalized Enrichment Score. **(C)** Fold increase in nerve associated genes such as NRG1, NRG2 and NGF (green) and fold decrease in atrophy genes Murf-1 and Atrogin-1 (red) after 5 days of dox-treatment, normalized to cells with no dox treatment.

Supplemental Figure 2: Heatmaps showing differentially expressed genes for key gene ontology biological process and cellular component pathways in TA muscle 5 weeks after nerve transection. (A) Synapse **(B)** Innervation **(C)** Distal Axon **(D)** Cellular response to PDGF stimulus **(E)** Semaphorin-Plexin signaling pathway involved in axon guidance **(F)** Nerve development **(G)** Skeletal System Development **(H)** Collagen Fibril Organization **(I)** Protein Polyubiquitination and **(J)** Autophagy. Only genes with statistically significant differences between WT and NANOG samples ($p<0.05$) are depicted. Genes of significant interest pertaining to synapse formation are highlighted by green arrows.

Supplemental Figure 3: GSEA analysis reveal key pathways upregulated by NANOG expression 5 week and 16 weeks after nerve transection. (A) Pie charts showing contribution of ECM-related pathways in WT and NANOG TA muscles after transection normalized to non-transected side. **(B)** Top 20 most highly upregulated GSEA pathways in WT mice 5 weeks after transection normalized to non-transected control limb. **(C)** Top 20 most highly upregulated GSEA pathways in NANOG mice 5 weeks after transection normalized to non-transected control limb. **(D)** Significantly upregulated GSEA pathways pertaining to ECM organization (red) and nerve regeneration (green) 16 weeks after transection. The FDR q-values for each pathway are depicted next to the bar; FDR q-value<0.25 has been considered significant.

Supplemental Figure 4: NANOG expression increases nerve myelination following nerve transection. Heatmaps showing differentially expressed genes for gene ontology pathways 5

weeks post nerve transection **(A)** Gliogenesis **(B)** Glial cell migration and **(C)** Myelin Assembly. Only genes with statistically significant differences between WT and NANOG samples ($p<0.05$) have been depicted. **(D)** Enrichment plot from Reactome database depicting enrichment of "EGR2 and Sox10 mediated initiation of Schwann Cell Myelination" pathway at 5 weeks post nerve transection. NES: Normalized Enrichment Score. **(E)** Immunocytochemistry for Fluoromyelin (red) in the transected nerve and control non-transected nerve of WT and NANOG animals 5 weeks post transection. Orange arrows represent proximal nerve stamp, green arrow represent distal nerve stamp, injury site is indicated in blue, white lines delineate the nerve in the image field. **(F)** High magnification images of each nerve at 5 weeks, showing that NANOG expression increases nerve myelination (red). **(G)** Quantification of mean grey intensity of fluoromyelin in injured and uninjured nerves of WT and NANOG mice.

Supplemental Figure 1

A)

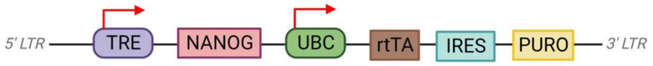

B)

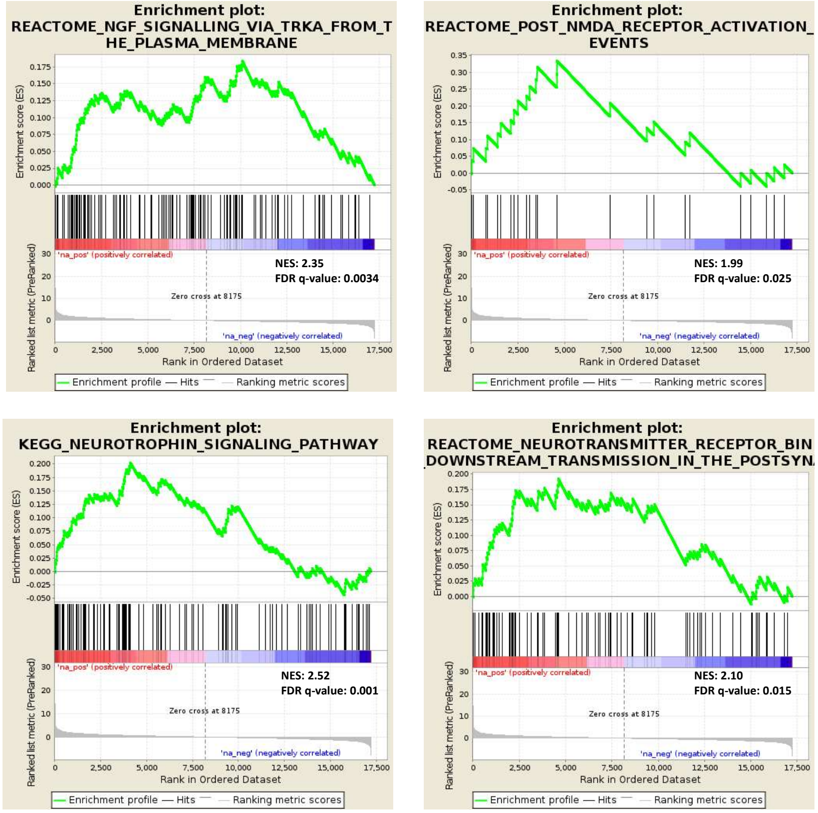

C)

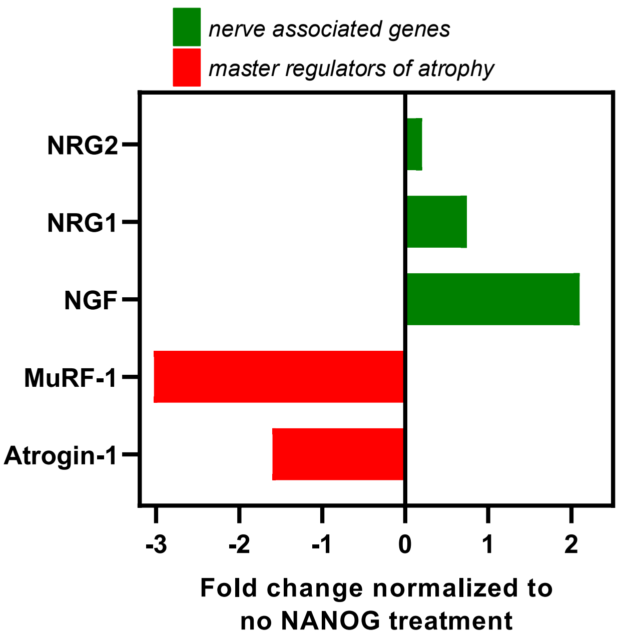

Supplemental Figure 2

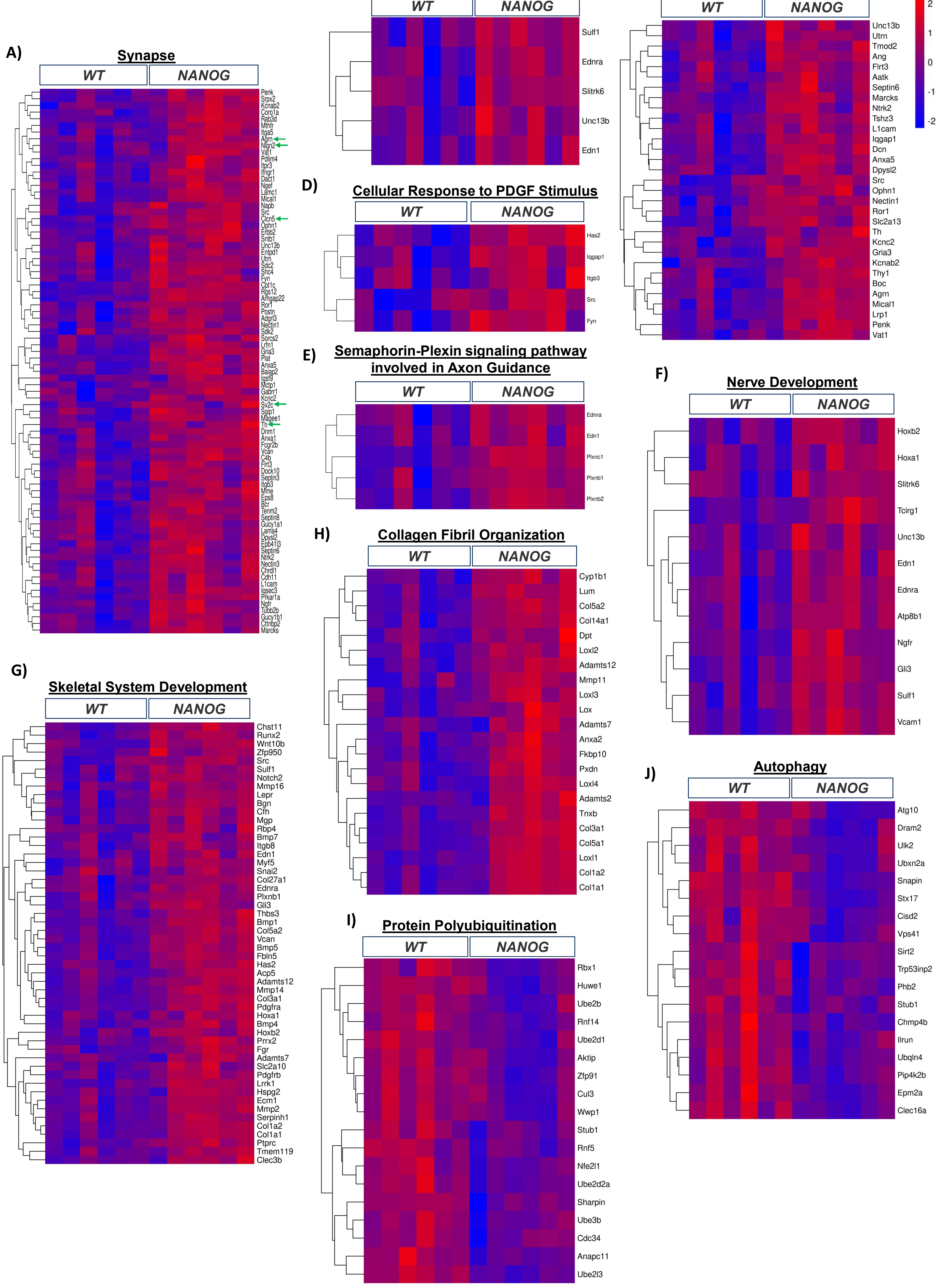

Supplemental Figure 3

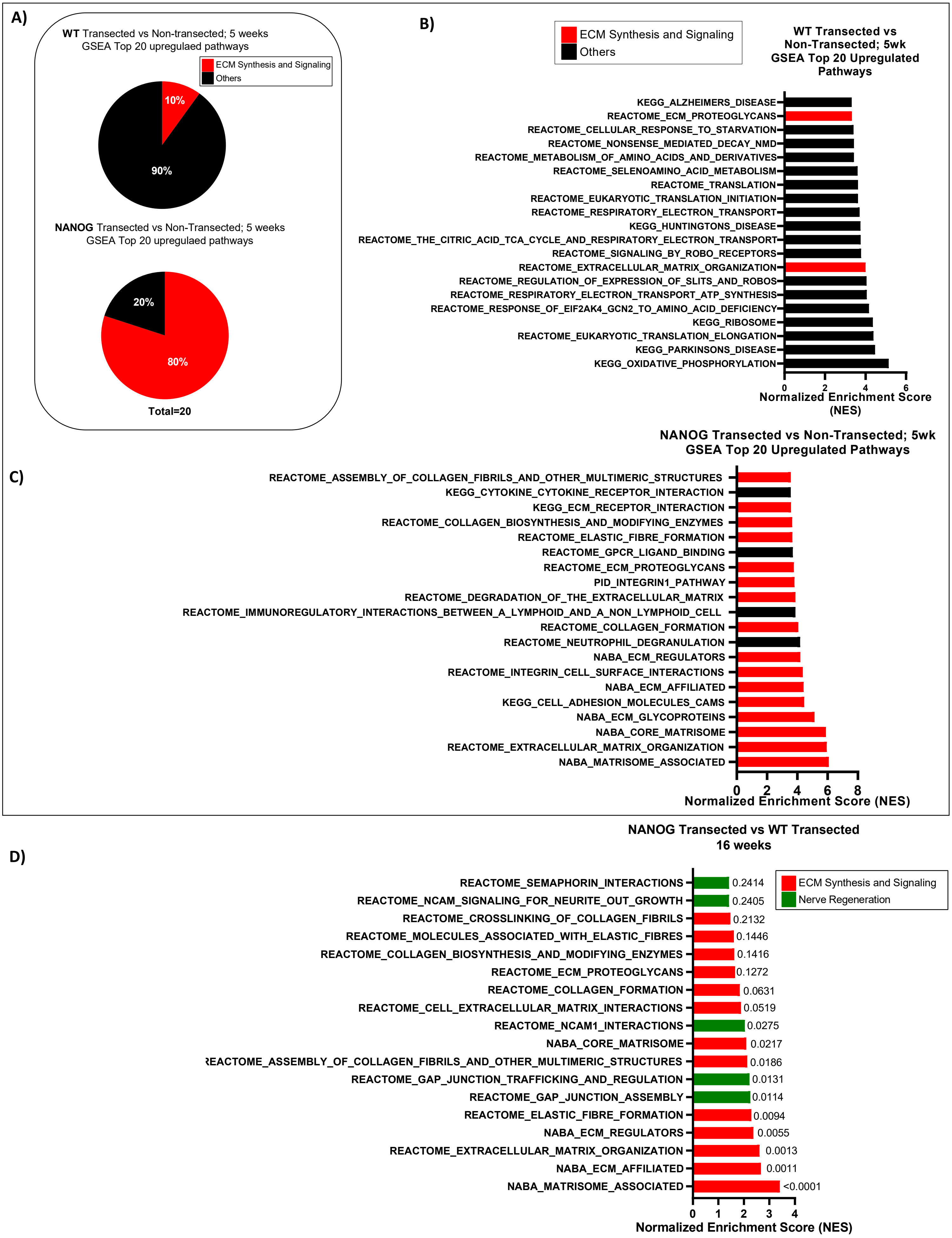

Supplemental Figure 4

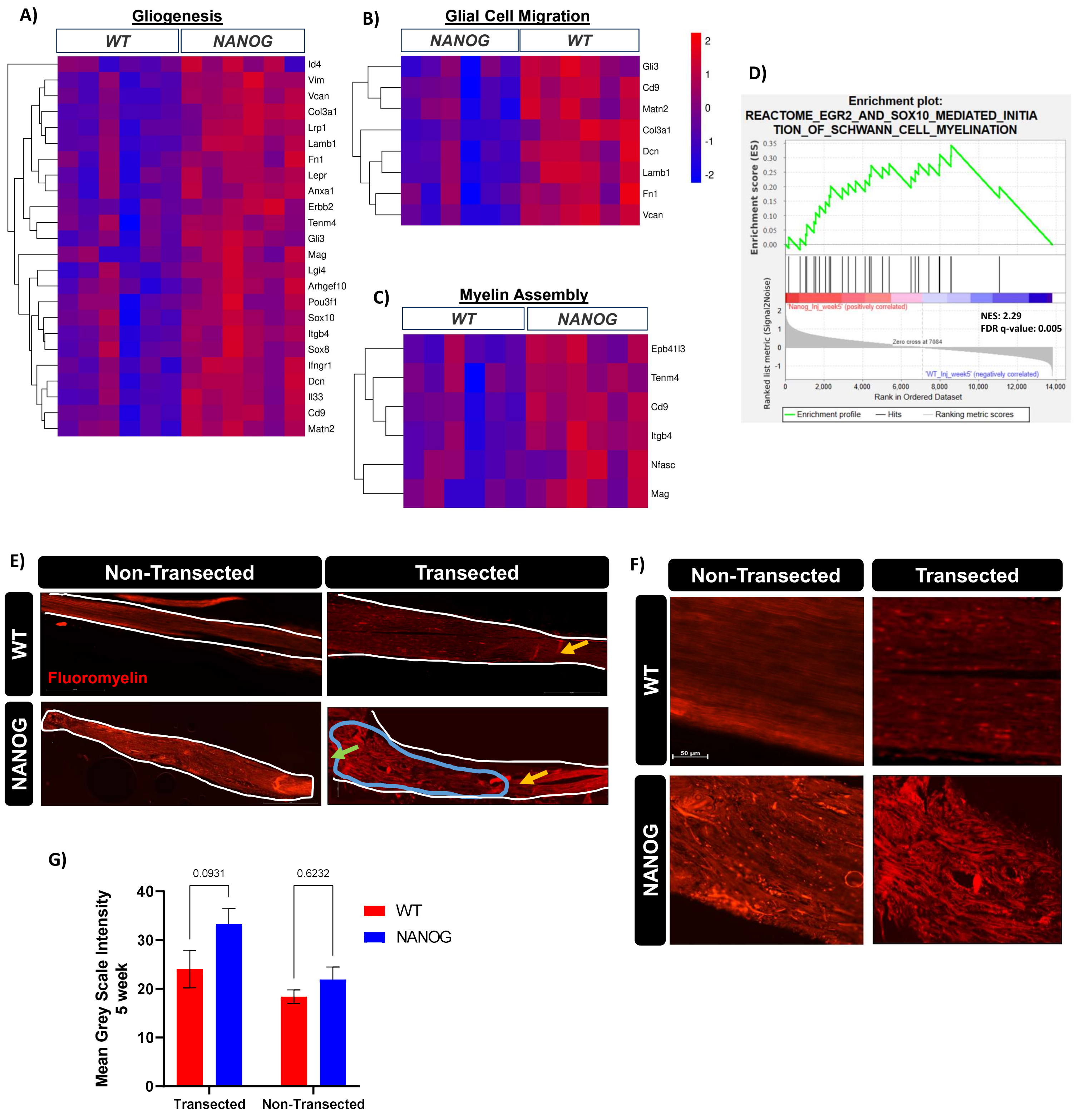

Supplement: Supplement 1 [file NIHPPRS3463557V1-supplement-1.pdf]
